# Human Coronavirus HKU1 Neutralizing Monoclonal Antibodies Target Diverse Epitopes Within and Around the TMPRSS2 Receptor Binding Site

**DOI:** 10.1101/2025.10.29.685445

**Published:** 2025-10-30

**Authors:** Lingshu Wang, Jeswin Joseph, Sheena Vasquez, Daniel Wrapp, Timothy P. Sheahan, Christian K.O. Dzuvor, Osnat Rosen, Robert N. Kirchdoerfer, Olubukola M. Abiona, Catherine Hammond, Wei Shi, Sydney P. Moak, Wing-Pui Kong, Yi Zhang, Michael R. Eso, Ariane J. Brown, Andrew B. Ward, Ralph Baric, Jason S. McLellan, Theodore C. Pierson, John Mascola, Barney S. Graham, Hadi M. Yassine, Christopher O. Barnes, Kizzmekia S. Corbett-Helaire

**Affiliations:** 1 Vaccine Research Center; National Institutes of Allergy and Infectious Diseases; National Institutes of Health; Bethesda, Maryland, 20892; United States of America; 2 Department of Immunology and Infectious Diseases; Harvard T.H. Chan School of Public Health; Boston, Massachusetts, 02115; United States of America; 3 Department of Biology; Stanford University; Stanford, CA, 94305; United States of America; 4 Department of Molecular Biosciences; University of Texas at Austin; Austin, Texas, 78712; United States of America; 5 Department of Epidemiology; University of North Carolina at Chapel Hill; Chapel Hill, North Carolina, 27599; United States of America; 6 Department of Integrative Structural and Computational Biology, The Scripps Research Institute, La Jolla, CA,92037; United States of America; 7 Howard Hughes Medical Institute; Chevy Chase, Maryland, 20815; United States of America; 8 Biomedical Research Center, Member of QU Health, Qatar University; Doha, Qatar; 9 ChEM-H Institute, Stanford University; Stanford, 94305, United States of America; 10 Chan Zuckerberg Biohub; San Francisco, 94158, United States of America; 11 Current affiliation: Duke Human Vaccine Institute, Duke University School of Medicine, Durham, NC 27710; United States of America; 12 Current affiliation: Department of Biotechnology, Israel Institute for Biological Research, Ness-Ziona, Israel; 13 Current affiliation: Department of Biochemistry, Institute for Molecular Virology, Center for Quantitative Cell Imaging, University of Wisconsin-Madison, Madison, Wisconsin, 53706; United States of America; 14 Current affiliation: Modex Therapeutics, Weston, MA, 02493; 15 Current affiliation: Medicine and Microbiology, Biochemistry, & Immunology, Morehouse School of Medicine, Atlanta, GA, 30310

## Abstract

Endemic human coronaviruses (HCoVs), like HCoV-HKU1, account for ~30% of common cold/year and can cause serious upper and lower respiratory infections, yet no licensed vaccines target HCoVs. In fact, little is known about HCoV-HKU1’s antigenic landscape. Thus, we characterized key interactions between HCoV-HKU1 spike (S) with monoclonal antibodies (mAbs) isolated from pre-pandemic HCoV-HKU1 convalescent PBMCs. We isolated 14 mAbs, which bound distinct S regions: receptor binding domain (RBD), N-terminal domain (NTD), and S2 subunit. Structural and functional studies revealed three groups of RBD-specific mAbs targeting diverse footprints within and around the TMPRSS2 receptor binding site, exemplified by: (1) The most potently neutralizing mAb, H501–022 (IC_50_ = 0.01 μg/mL), which recognizes the TMPRSS2 binding motif, thereby blocking receptor engagement; (2) mAb H501–008 (IC_50_ = 0.05 μg/mL) that binds a conserved, cross-reactive epitope outside of the TMPRSS2 binding site that is shared with HCoV-OC43; and (3) H501–018 (IC_50_ = 0.28 μg/mL) that recognizes both “up” and “down” RBD conformations at a distinct, non-overlapping site outside of the TMPRSS2 binding motif, distinguishing itself from H501–022 and H501–008, which bind exclusively to the “up” RBD conformation. These mAbs represent the first type-specific HCoV-HKU1 mAbs isolated from a convalescent donor. Our findings provide molecular insight into HCoV-HKU1 antibody recognition and neutralization mechanisms, importantly highlighting antigenic differences comparing HCoVs and pandemic CoVs – a critical step towards advancing universal CoV vaccine design.

## Introduction

COVID-19, disease caused by severe acute respiratory syndrome coronavirus (SARS-CoV-2), has claimed over seven million lives globally, emphasizing coronaviruses (CoVs) are dire global pandemic threats^1^. However, there are four endemic human coronaviruses (HCoVs), which are classified into alpha (HCoV-229E and HCoV-NL63) and beta (HCoV-HKU1 and HCoV-OC43) genera and are responsible for up to 30% of seasonal common colds^2^. HCoV infection typically manifests as either asymptomatic or mild upper respiratory disease^3^, but can also result in hospitalization and death, particularly in frail or co-morbid individuals or with co-infections^3–6^.

Despite widespread circulation, there are no licensed vaccines or therapeutics for HCoVs, although the idea of combining HCoV antigens into supra-seasonal or universal CoV vaccines has been explored in pre-clinical studies by our group and others^7^. Unfortunately, such vaccine development is hindered by incomplete understanding of the antigenic landscape of HCoV major surface protein, spike (S). For Middle East respiratory syndrome (MERS)-CoV, SARS-CoV, and SARS-CoV-2, S particularly, the receptor binding domain (RBD), is the principal target of potently neutralizing antibodies and is thus the preferred CoV vaccine antigen^8^. RBDs are in the C-terminal domain of the S1 head region and play a critical role in viral entry^9,10,^ whereas N-terminal domain (NTD) typically mediates interactions with accessory host cellular attachment factors^10,11^.

Until recently, *O*-acetylated sialic acids had been pinpointed as attachment factors for beta-HCoVs^12^, but no functional entry receptors had been discovered. A series of recent studies delineated the two-step receptor engagement process of HCoV-HKU1 S^13, 14^. First, HCoV-HKU1 NTD binds to cellular sialic acid moieties, which triggers RBD exposure^13^. Next, HCoV-HKU1 RBD binds to the functional entry receptor, transmembrane protease, serine 2 (TMPRSS2)^14^. The glycoprotein interaction, followed by conformational changes in RBD, enables viral-host membrane fusion and subsequent HCoV-HKU1 infection. Structural studies of HCoV HKU1 S revealed the occluded nature of RBD^10^, compared to epidemic CoVs which have transient open-close RBD conformations^11,15^. RBD occlusion may represent an immune evasion mechanism adapted by HCoV-HKU1, shielding its immunodominant RBD domain. Similar immune evasion mechanisms have been reported in human immunodeficiency virus (HIV), wherein HIV uses a primary receptor to induce conformational changes followed by secondary receptor interaction^16^.

Delineating prevalence of diverse monoclonal antibodies (mAbs) following HCoV-HKU1 infection could reveal the antigenic landscape of the open/closed state of S. Extensive global efforts have led to isolation and structural characterization of hundreds of potently neutralizing mAbs targeting diverse S epitopes on epidemic CoVs, particularly within RBD and NTD^17,18^. These discoveries revealed key mechanisms of viral neutralization, identified broadly cross-reactive antibody lineages and directly informed design of antibody-based therapeutics and next-generation vaccines. In contrast, such systematic mAb discovery for endemic HCoVs, like HCoV-HKU1, remains scarce. In fact, only recently has a HCoV-HKU1 RBD mAb (R7) been described, albeit isolated via a phage display platform^19^. In all, significant gaps in understanding HCoV-HKU1’s antigenic landscape remain, for which improved knowledge could be leveraged for designing vaccines with broader CoV protection.

Here, we utilized human HCoV-HKU1-positive peripheral blood mononuclear cells (PBMCs), acquired prior to the COVID-19 pandemic and a rational B cell sorting strategy to isolate a panel of mAbs. The resulting 14 mAbs target distinct S protein regions: RBD (N = 11), NTD (N = 2), and S2 stem (N = 1) domains. Anti-HKU1 RBD-specific mAbs were categorized into three major competition groups, suggesting multiple mechanisms of action. Structures of neutralizing anti-HKU1 RBD-specific mAbs from each competition group bound to prefusion-stabilized HCoV-HKU1 S (S-2P)^20^ revealed discrete sites of vulnerability, including an HCoV-OC43 cross-reactive epitope outside of the TMPRSS2 binding motif, and provide a molecular framework for understanding neutralizing antibody mechanisms of HCoVs. Together, this work presents isolation and characterization of the first type-specific HKU1 mAbs from a convalescent donor, describes antigenic features of HCoV-HKU1 S, and will enable better appreciation of differences and similarities between epidemic and endemic HCoVs. In the context of universal CoV vaccine development, these results will prove beneficial towards assessing vaccine-elicited immune responses, increasing understanding of immune imprinting, and, importantly, broadening immunological reach of antigen designs.

## Results

### HCoV-HKU1-positive convalescent PBMCs yield mAbs that bind various S domains

Human donor “A” from VRC-200 clinical study presented with acute HCoV-HKU1 infection in 2014 (5 years prior to the COVID-19 pandemic); sera and PBMCs were collected during early convalescence^21^. Serum collected 41 days after HCoV-HKU1 diagnosis reacted to both HCoV-HKU1 and -OC43 S proteins, but not SARS- or MERS-CoV, due to probable repeated HCoV infections throughout the donor’s lifetime^22^ (Extended Data Fig.1). Therefore, we isolated mAbs from B cells using HCoV-HKU1 S-2P probes, and probe-positive cells were further subjected to sequencing for identification of antibody heavy and light chain sequences (Supplementary Fig. 1 and Table 1). Fourteen mAbs (H501–003, -007, -008, -009, -010, -012, -013, -014, -015, -016, -018, -020, -022, and -101) were isolated.

First, to determine S region binding specificity, we assessed the mAbs’ ability to bind HCoV-HKU1 S-2P^10^, S1, and RBD proteins via ELISA, and eleven mAbs (H501–003, -008, -009, -012, -013, -014, -015, -016, -018, -020, and -022) specifically bound to HCoV-HKU1 RBD. Two mAbs (H501–007 and -010) bound to HCoV-HKU1 S-2P and S1, but not RBD, and were thus determined to be NTD-specific. One mAb (H501–101) showed positive binding to HCoV-HKU1 S-2P, but not to S1 or RBD, so it was determined to be S2-specific (Table 1 and Extended Data Fig. 2). With exception of H501–003, -007, and -018, the mAbs bound equally to HCoV-HKU1 S-2P with half-maximal effective concentration (EC_50_) values of less than 0.01 μg/ml (Table 1 and Extended Data Fig. 2). Similarly, binding affinities, as determined by biolayer interferometry (BLI), showed high affinity interactions of the mAbs bound to their corresponding HCoV-HKU1 S domains with H501– 020 and -101 showing the highest affinities (<1 pM) to HCoV-HKU1 RBD and S-2P proteins, respectively. (Table 1 and Extended Data Fig.3). RBD mAbs generally showed higher affinity compared to NTD mAbs (Table 1).

**Figure 1.**
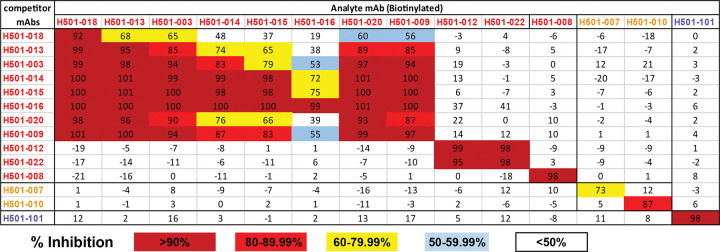
HCoV-HKU1 mAb competition map. HCoV-HKU1 RBD- (red), NTD- (orange), and S2-specific (purple) mAbs were biotinylated and assessed for competition binding to HCoV-HKU1 S-2P bound to mAbs listed in the leftmost column. Analyses were completed by ELISA. Percent inhibition of analyte (biotinylated)-mAb binding by competitor (unlabeled) mAb is indicated by color. Dark red = >90% competition, red = 80–99.99% competition, yellow = 60–79.99% competition, light blue = 50–59.99% competition, and uncolored = <50% competition.

Next, we evaluated competitive binding of biotinylated anti-HKU1 mAbs to HKU1-S-2P using ELISA (Fig. 1) and BLI (Extended Data Fig. 4). RBD mAbs H501–012 and -022 showed competitive binding to HKU1-S-2P, consistent with their close clonal relationship (i.e., sharing the same heavy and light chain germline genes, identical CDRH3 and CDRL3 lengths, and >90% identity across both regions) (Fig. 1, Extended Data Fig. 4, and Supplementary Table 1). H501–008 exhibited a unique binding pattern, showing no competition with other RBD mAbs, indicating it targets a distinct epitope. All other RBD mAbs, with exception of H501–018, displayed varying degrees of competition, suggesting a closely localized binding epitope. In fact, H501–018 showed limited competition with other RBD mAbs. NTD mAbs, H501–007 and -010, competed with each other but not with RBD or S2 mAbs. S2-specific mAb H501–101 showed no competition with RBD mAbs or NTD mAbs (Fig. 1 and Extended Data Fig. 4). Based on these competition profiles, RBD-specific mAbs segregated into three distinct groups: one containing mAb H501–008, a second comprising mAbs H501–009, -018, -013, -003, -014, -015, -016, and -020, and a third including mAbs H501–012 and -022. Consistent with previous studies^23,24^, our data suggest CoV infection elicits mAbs targeting diverse S regions with varying affinities. However, it is important to determine the mAbs’ neutralization potential to provide insights into potential mechanisms of action and downstream targets of vaccine antigen design.

### Three HCoV-HKU1 RBD-specific mAbs are potently neutralizing

To that end, we evaluated neutralization potential using HCoV-HKU1 pseudoviruses (genotype C, a lineage that emerged in 2006)^25^. HEK293T cells expressing human TMPRSS2 were infected with HCoV-HKU1 pseudoviruses complexed with serial diluted mAbs. RBD mAbs demonstrated varying degrees of neutralization against HCoV-HKU1, whereby H501–008, -012, and -022 were the most potent with half-maximal inhibitory concentrations (IC_50_) values of 0.05, 0.02, and 0.01 μg/mL, respectively. The other RBD mAbs had little to no neutralization capacity against HCoV-HKU1 with IC_50_ values ranging from 0.28 – 4.91 μg/mL. In contrast, none of the NTD or S2 mAbs were able to effectively neutralize HCoV-HKU1 pseudoviruses (IC_50_ > 10 μg/mL) (Table 1). Although there are several notable exceptions^26^, CoV NTD and S2 mAbs are generally moderately neutralizing or non-neutralizing^27,28^, just as we observe here. Importantly, though, these neutralization data show HCoV-HKU1 RBD mAbs can be potently neutralizing. This phenomenon is common to CoV mAbs, whereby RBD is immunodominant and elicits potential neutralizing antibodies, hence RBD often being the target of CoV vaccine designs^27,28^. In fact, we recently designed and evaluated HCoV-HKU1 immunodominant domain vaccine antigens, consisting of convergent RBD sequences, based, in part, on our knowledge of the potency of HCoV-HKU1 RBD mAbs described herein^21^.

### Potently neutralizing RBD mAb H501–008 and S2-specific mAb H501–101 cross-react with HCoV-HKU1 and -OC43

**Figure 2.**
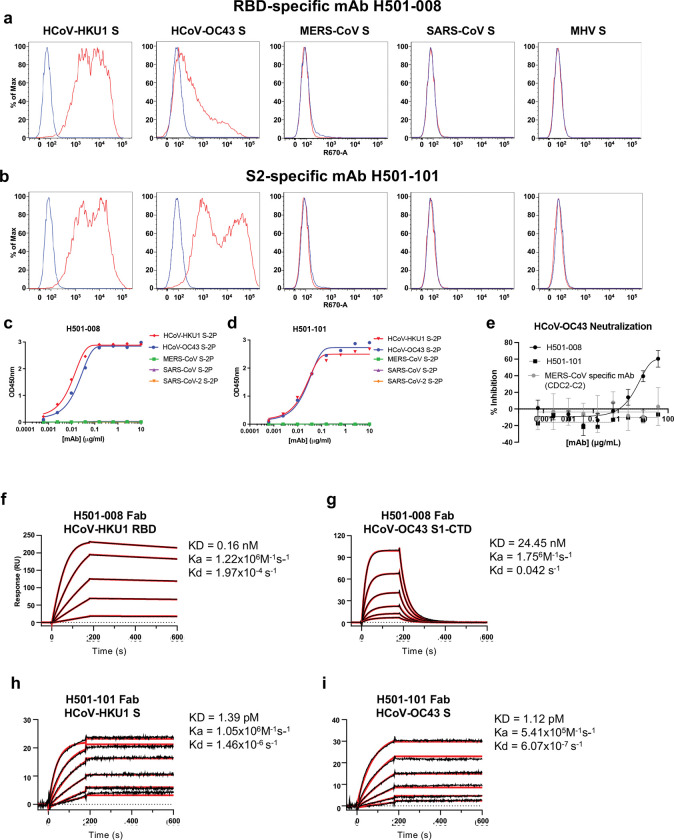
An RBD- and a S2-specific mAb cross-react with HCoV-HKU1 and -OC43. (a-b) RBD-specific mAb, H501–008 (a), and S2-specific mAb, H501–101 (b), were assessed for binding to cell surface-expressed S proteins from HCoV-HKU1, HCoV-OC43, MHV-CoV, MERS-CoV, and SARS-CoV. The percentage of maximum binding to S-transfected cells (red) compared to untransfected cells (blue) are shown. (c-d) H501–008 (c) H501–101 (d) binding to soluble HCoV-HKU1 (red), HCoV-OC43 (blue), MERS-CoV (green), SARS-CoV (purple), and SARS-CoV-2 (orange) S-2P proteins was assessed by ELISA. (e) The ability of H501–008 (black circles) and H501–101 (black squares) to neutralize native HCoV-OC43 was assessed. MERS-CoV specific mAb, CDC2-C2 (gray) was used as a negative control. Error bars represent standard deviation of three individual measurements. (f-g) Binding affinities of H501–008 Fabs bound to HCoV-HKU1 (f) and HCoV-OC43 (g) S1-CTD as assessed by surface plasmon resonance. (h-i) Binding affinities of H501–101 Fabs bound to HCoV-HKU1 (h) and HCoV-OC43 (i) S as assessed by surface plasmon resonance.

Because the donor’s serum bound to both HCoV-HKU1 and -OC43 S proteins, we analyzed cross-reactivity of the 14 isolated mAbs to S proteins from other beta-CoVs, HCoV-OC43, MHV, MERS-CoV, SARS-CoV, and/or SARS-CoV-2. Interestingly, RBD-specific H501–008 and S2-specific H501–101 cross-reacted with cell surface-expressed HCoV-HKU1 and -OC43 S (Fig. 2a,b) and soluble S via ELISA (Extended Data Fig. 5 and Fig. 2c,d). Further, we tested these two mAbs’ abilities to neutralize authentic HCoV-OC43 virus and found H501–008 weakly neutralizes HCoV-OC43 (IC_50_ > 10 μg/mL); H501–101 does not neutralize at all (Fig. 2e). The mAbs’ lack of cross-reactivity with epidemic CoVs is to be expected since the PBMCs were acquired prior to SARS-CoV-2 emergence, and the donor most likely had not been previously exposed to SARS- or MERS-CoV.

We next performed surface plasmon resonance (SPR) experiments to more thoroughly characterize binding kinetics of H501–008 and -101 to HCoV-HKU1 and -OC43 RBDs. H501–008 Fab bound to HCoV-HKU1 RBD with subnanomolar affinity (161.0 pM) and to HCoV-OC43 RBD, albeit with reduced affinity (~25 nM) that can primarily be attributed to an increased off-rate (Fig. 2f,g). Similarly, H501–101 Fab bound with nearly equal affinities of 1.39 pM and 1.12 pM to HCoV-HKU1 and -OC43, respectively (Fig. 2h,i). The cross-reactive epitope highlighted by S2 mAb H501–101 is plausible as HCoV-HKU1 and -OC43 S2 share ~72% amino acid similarity. Additionally, a plethora of cross-reactive CoV S2-specific mAbs have been isolated to date^29–31^. Beyond S2, these data allude to a cross-reactive epitope within HCoV-HKU1 and -OC43 RBD, which is interesting due to HCoV-HKU1’s receptor being TMPRSS2, while cell surface 9-*O*-acetylated sialic acid has been coined the receptor for HCoV-OC43^12^.

### HCoV-HKU1/OC43 RBD mAb, H501–008, binds outside of TMPRSS2 binding site and traps RBD in “up” confirmation

**Figure 3.**
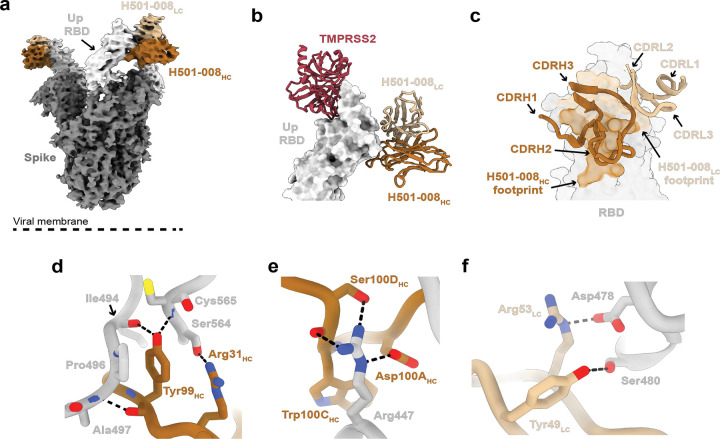
Structure of HCoV-HKU1 S-2P bound to H501–008. (a) Cryo-EM map of HCoV-HKU1 S-2P bound to H501–008 (b) TMPRSS2 (PDB 8Y8B) is aligned based on the RBD bound to H501–08 V_H_ and V_L_ from crystal structure. RBD is in a surface representation, and TMPRSS2 and H501–008 are displayed as ribbons. (c) Close-up view of the H501–008 mediated contacts on the RBD epitope, colored by the V_H_ and V_L_ domain contributions. (d-e) Interacting residues between H501–008 heavy chain and HCoV-HKU1 C RBD. (f) Interacting residues between the H501–008 light chain and HCoV-HKU1 RBD. Residues are shown as sticks. Colors are as follows: Spike (gray), RBD (light gray), H501–008 V_H_ (orange), H501–008 V_L_ (tan), TMPRSS2 (red).

To that end, we set out to characterize molecular determinants that mediate H501–008 cross-reactivity using cryo-EM studies of H501–008 Fab in complex with the HCoV-HKU1 S-2P (Extended Data Fig. 6). Remarkably, we observed HCoV-HKU1 RBD trapped in the rarely observed “up” conformation by the H501–008 Fab. Similar to RBD conformations observed in related betacoronaviruses, such as MERS- and SARS-CoV^20,32,33^, HCoV-HKU1 RBD juts up away from the rest of the S, exposing the S2 domain heptad repeat domain 1 helices (Figure 3a). While we observed density for H501–008 Fabs bound to the “up” RBDs, conformational plasticity prevented us from solving the structure to high-resolution. Thus, we performed X-ray crystallographic studies of the H501–008 Fab bound to the isolated HCoV-HKU1 RBD domain and solved the complex structure to a resolution of 2.60 Å (Supplementary Table 2). Despite high-resolution of this dataset, there was only a single Fab + RBD complex in the asymmetric unit, leading to an extremely high solvent content (~70%) that resulted in relatively poor electron density for some map regions, limiting model building. Electron density at the interface between the H501–008 CDRs and the HCoV-HKU1 RBD was interpretable, and the Fab binding orientation agreed well with the results of our comparatively low-resolution cryo-EM map (Figure 3a,b).

Analysis of our hybrid structural studies revealed several key contacts that are distributed across the CDRH1, CDRH3 and CDRL2 loops (Figure 3c). Key contacts include potential hydrogen bonds between Arg31_HC_, the side chain of Ser564, and the backbone amine of Cys565 hydrogen bonding with Tyr99_HC_, which simultaneously binds to the backbone carboxyl of Ile494 (Figure 3d). In addition to Tyr99_HC_, CDRH3 residues Asp100_A-HC_ and Ser100_D-HC_ hydrogen bond with Arg447 via their side chains (Figure 3e). H501–008 light chain interactions are mostly confined to the CDRL2, where Tyr49_LC_ and Arg53_LC_ hydrogen bond with the side chain of Ser480 and Asp478, respectively (Figure 3f).

By comparing key contact residues in HCoV-HKU1 RBD to their counterparts in the HCoV-OC43 RBD, we can begin to understand molecular determinants that permit H501–008 cross-reactivity (Extended Data Fig. 7). HCoV-HKU1 RBD shares 53.2% sequence identity with HCoV-OC43 RBD over the 297 residues that we recombinantly expressed for crystallization. Given this relatively minimal degree of sequence conservation, it is perhaps surprising that the humoral immune response could elicit an Ab that recognizes both HCoV-HKU1 and -OC43 RBDs. For example, several key residues that mediate H501–008 binding, such as Arg447 and Ala497, are strictly conserved in HCoV-OC43 as Arg463 and Ala524, respectively (Extended Data. Fig. 7h). The diminished binding affinity observed with the HCoV-OC43 RBD is likely due, in part, to an insertion in the HCoV-HKU1 RBD at positions 558–565. Although additional contacting residues differ between the two RBDs (e.g., Ile494), these variations likely have a lesser impact on binding, as corresponding H501–008 residues interact primarily with the peptide backbone, which remains structurally invariant despite sidechain differences.

Further, alanine mutation scanning of HCoV-HKU1 and HCoV-OC43 S proteins followed by a cell surface mAb binding assay revealed that amino acid residues Ala497 and Arg447 are critical for H501–008 binding to HCoV-HKU1 S (Extended Data. Fig. 8a). Similarly, Ala524 and Arg463 are important for H501–008 interaction with the OC43 S (Extended Data. Fig. 8b). Pseudovirus neutralization assay of HCoV-HKU1 S mutants confirmed cell surface staining data, emphasizing the importance of A497 in H501–008 interaction with HCoV-HKU1 S (Extended Data. Fig. 8c). R447A mutant pseudovirus was less infectious in HEK293T-TMPRSS2 cells, potentially due to conformational changes in the S RBD (Supplementary Fig.2).

### HCoV-HKU1 RBD mAb, H501–018, recognizes RBD in two conformational states

**Figure 4.**
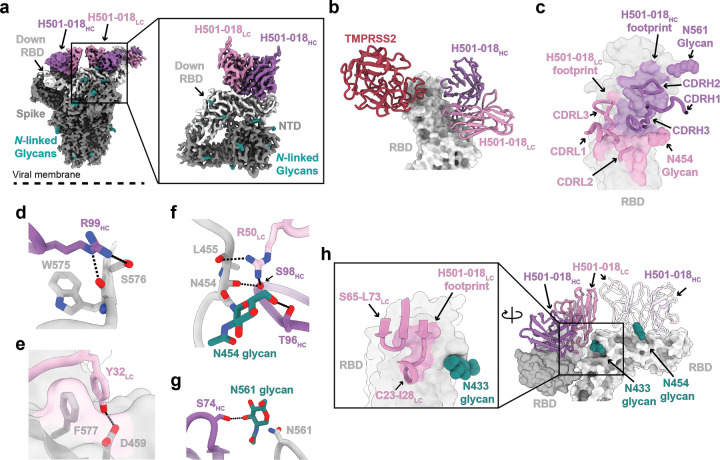
Structure of HCoV-HKU1 S-2P bound to H501–018. (a) Cryo-EM map of HCoV-HKU1 S-2P bound to H501–018 from State 2. Inset shows the locally refined map of the V_H_ and V_L_ domains, the RBD, and the NTD. (b) TMPRSS2 (PDB 8Y8B) is aligned based on the RBD bound to H501–018 V_H_ and V_L_ from the locally refined cryo-EM structure. RBD is displayed as a surface representation, and TMPRSS2 and H501–018 are displayed as ribbons. (c) Close up view of the H501–018 mediated contacts on the RBD epitope colored by the V_H_ and V_L_ domain contributions. (d-g) Interacting residues between H501–018 and HCoV-HKU1 RBD. Residues are shown as sticks. (h) H501–018 bound to a “down” RBD in State 2 contacting a neighboring RBD. Inset shows the RBD epitope from the neighboring H501–018 light chain. Colors are as follows: S (gray), RBD (light gray), H501–018 V_H_ (purple), H501–018 V_L_ (pink), *N*-linked glycans (teal), TMPRSS2 (red).

To investigate structural basis of the potently neutralizing mAb H501–018 (Figure 1), we determined a 3.4 Å resolution single-particle cryo-EM structure of HCoV-HKU1 S-2P bound to H501–018 Fab. We revealed two different conformational states for the H501–018 Fab – S-2P complex. In State 1, two RBDs adopt the “up” conformation, while one remains “down” (Extended Data. Fig. 6b), whereas in State 2, only a single RBD adopts the “up” conformational state (Fig. 4a). To better resolve the molecular interactions at the Fab – RBD interface, we performed local classification and refinement on cryoEM density corresponding to H501–018 Fab bound to a “down” RBD. This yielded a locally refined density map to 3.2 Å resolution, allowing for modeling of the H501–018 – RBD complex (Fig. 4a)^34^. Our structure revealed a binding orientation for H501–018 that engages an RBD epitope that does not overlap with the TMPRSS2 receptor binding motif (Fig. 4b), suggesting that its mechanism of neutralization does not involve direct competition with TMPRSS2.

Analysis of the Fab – RBD interface revealed that all H501–018 CDR loops contact RBD, with an epitope buried surface area (BSA) of 1,064 A^2^ (551 Å^2^ BSA against the heavy chain; 513 Å^2^ against the light chain) (Fig. 4b,c)^35^. Critical contacts involve a combination of germline-encoded and somatically hypermutated H501–018 residues including: Arg99_HC_ hydrogen bonding to the backbone carboxyl of Trp575 and the sidechain of Ser576 (Fig. 4d); Tyr32_LC_ of the CDRL1 loop forming a pi-stacking interaction and hydrogen bonding with Phe577 and Asp459 in the RBD, respectively (Fig. 4e); and, Arg50_LC_ forming a hydrogen bonding network at the base of the Asn454-glycan (Fig. 4f). Ser98 also engages in hydrogen bonding with the backbone carboxyl of Asn454, which would have possibly been hindered if Ser98 did not undergo somatic hypermutation from a previous Trp98^36,37^. Additionally, we observed two glycan-mediated interactions between the heavy chain and RBD: Thr96_HC_ of the CDRH3 loop forms a hydrogen bond with the Asn454-glycan (Fig. 4f), an interaction mediated by Trp98Ser_HC_ somatic mutation to accommodate glycan recognition, and another between the Asn561-glycan and Ser74_HC_ (Fig. 4g). Interestingly, in the State 2 complex, H501–018 framework regions 1 and 3 engage with “down” RBD of the neighboring protomer establishing a quaternary epitope that likely contributes to increased affinity of the Fab at trimer interface in this state (Fig. 4h).

### HCoV-HKU1 mAb, H501–022, binds a neutralization-sensitive TMPRSS2-blocking epitope

**Figure 5.**
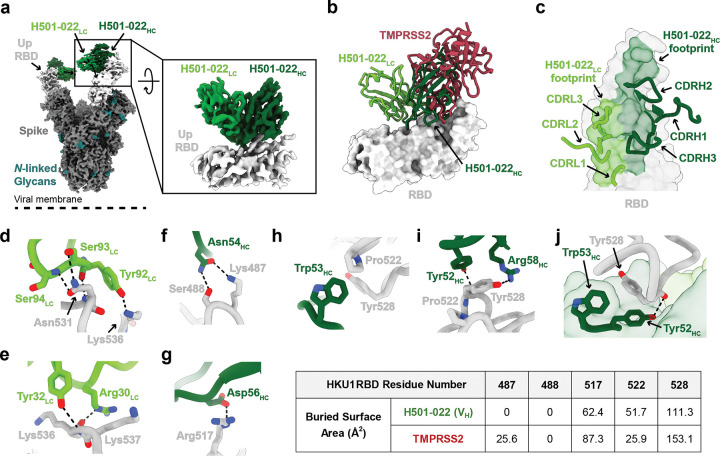
Structure of HCoV-HKU1 S-2P bound to H501–022. (a) Cryo-EM map of HCoV-HKU1 S-2P bound to H501–022. Inset shows the locally refined map of the V_H_ and V_L_ domains, and the S1 RBD.(b) TMPRSS2 (PDB 8Y8B) is aligned based on the RBD bound to H501–022 V_H_ and V_L_ from the locally refined cryoEM structure. (c) Close up view of the H501–022 mediated contacts on the RBD epitope colored by the V_H_ and V_L_ domain contributions. H501–022 CDR loops are shown as ribbons, and the RBD is a surface representation. (d-e) Interacting residues between H501–022 light chain and HCoV-HKU1 RBD. (f-j) Interacting residues between H501–022 heavy chain and HCoV-HKU1 RBD. The table shows the Buried Surface Area (BSA) for residues on the HCoV-HKU1 RBD contributed by H501–022 heavy chain or TMPRSS2. Colors are as follows: S (gray), RBD (light gray), H501–022 V_H_ (green), H501–022 V_L_ (light green), *N*-linked glycans (teal), TMPRSS2 (red).

To elucidate the molecular mechanism by which H501–022 binds RBD and blocks HCoV-HKU1 pseudovirus entry, we determined the 3.6 Å single-particle cryoEM structure of the HCoV-HKU1 S-2P bound to H501–022 Fabs (Fig 5a, Extended Data Fig. 6c, Supplementary Table 3). Global maps revealed all three RBDs adopting the “up” conformation and bound to H501–022 Fab (Fig. 5a). Given structural plasticity of the “up” conformation, local classification and refinement was performed at the Fab – RBD interface to generate cryoEM maps suitable for model building and refinement (Fig. 5b, Extended Data Fig. 6c, Supplementary Table 3). H501–022 adopts a binding orientation that overlaps with the RBD TMPRSS2 receptor binding motif, suggesting a neutralization mechanism that directly inhibits virus engagement with cell surface TMPRSS2 (Fig. 5b). While all CDR loops engage the epitope, majority of the interactions are contributed by the CDRH2 and CDRH3 loops, which contribute 31.8% and 21.5% of the total paratope BSA, respectively (Fig. 5c)^35^. Although both chains contribute to the interaction, the light chain engages the TMPRSS2 binding site to a lesser extent than the heavy chain (Fig. 5c). Within the light chain interface, CDRL3 residues engage a patch on the RBD centered around Asn531 and Lys536. Tyr92_LC_ forms hydrogen bonds with both the backbone and side chain of Asn531, while nearby residues Ser93_LC_ and Ser94_LC_ contribute additional hydrogen bonding via backbone contacts (Fig. 5d). Tyr32_LC_ and Arg30_LC_ further stabilize the interaction by contacting Lys536 and Lys537, respectively (Fig. 5e).

The CDRH2 loop plays a critical role in blocking TMPRSS2. It engages several RBD residues previously identified as TMPRSS2 contact points, including Lys487, Arg517, Pro522, and Tyr528 (Fig. 5f–j, Extended Data Figure 7d–g.)^38,39^. Asn54_HC_ forms hydrogen bonds with Lys487 and Ser488, while Asp56_HC_ forms a hydrogen bond with Arg517 (Fig. 5f,g). Trp53_HC_ stacks against Pro522, while Tyr52_HC_ and Arg58_HC_ form hydrogen bonds with Tyr528 (Fig. 5h–j). Importantly, Tyr528 lies within a pocket at the V_H_ domain interface, contributing 111 Å^2^ BSA (Fig. 5n). Supporting the structural dataset, cell surface binding assays showed H501–022 blocked HCoV-HKU1 S binding to cell surface-expressed TMPRSS2 (Extended Data. Fig. 9a,b), and this extensive Tyr528 interaction helps explain why mutating Tyr528 to alanine substantially reduced H501–022 binding to the cell surface-expressed HCoV-HKU1 S (Extended Data. Fig. 9c). Further, multiple sequence alignment of HCoV-HKU1 S protein sequences from genotype A, B and C demonstrates that H501–022 critical binding residues are well conserved across HCoV-HKU1 strains (Supplementary Fig. 3), suggesting conservation in the TMPRSS2 binding site and moreover that H501–022 is likely broadly neutralizing across HCoV-HKU1 strains. Collectively, structures of RBD-specific mAbs from the three major competition groups bound to HCoV-HKU1 S provide the molecular framework for understanding neutralizing antibody recognition of endemic beta-HCoVs (Extended Data Fig. 10).

## Discussion

HCoVs represent a diverse group of respiratory viruses with varied clinical impacts^40^. While epidemic CoVs, such as SARS-CoV-2, have caused deadly outbreaks, four endemic HCoVs, HCoV-229E, -NL63, -OC43, and -HKU1, circulate globally typically causing mild respiratory disease, particularly in children. Elderly, immunocompromised, and otherwise co-morbid individuals are at higher risk for severe HCoV disease^41^. Despite broad circulation and contribution to global disease burden, endemic HCoVs remain understudied, particularly towards vaccine and therapeutic development. This is especially concerning for many reasons. Firstly, HCoV co-infection with other respiratory viruses impacts disease severity^3^. As well, HCoV infections potentially influence immune imprinting over one’s lifetime. Moreover, HCoV prevalence may shape emergence potential of other CoVs.

Herein, we address a critical knowledge gap of humoral immunity to endemic HCoVs by isolating and characterizing mAbs from a pre-COVID-19 pandemic HCoV-HKU1 convalescent donor. The 14 identified mAbs recognize diverse antigenic sites on HCoV-HKU1 S, the key surface glycoprotein responsible for receptor binding and membrane fusion. Structural analyses revealed three distinct antibody footprints within and around the TMPRSS2 receptor binding site, a region essential for viral attachment and entry into host cells (Extended Data. Fig. 10). Importantly, RBD-specific mAbs, H501–012 and H501–022, exhibited potent neutralization by directly blocking TMPRSS2 engagement, a mechanism reminiscent of SARS-CoV-2 Class 1 RBD mAbs that bind “up” RBD conformation and block S–ACE2 receptor interactions^33,42^. Recently, during preparation of our manuscript, another group described a HCoV-HKU1 RBD mAb (R7) through a phage display platform that overlaps with the TMPRSS2 binding motif. However, said orthogonal study used recombinant RBD for structural characterization and thus could not elucidate information about recognition of up/down RBD conformations. Additionally, R7’s ability to neutralize HCoV-HKU1 was undetermined^19^. Furthermore, we isolated our mAbs from a pre-COVID-19 pandemic convalescent donor, which provides better understanding of natural immune responses and insights into the antigenic landscape of HCoV-HKU1 S.

Characterization of H501–008 revealed potent neutralization of HCoV-HKU1 and cross-reactivity, though weak neutralization with HCoV-OC43, mapping to a conserved RBD epitope that may confer broader HCoV recognition. H501–008-like mAbs resemble Class V SARS-CoV-2 RBD-specific antibodies that bind exclusively to the “up” RBD conformation and exhibit broad sarbecovirus reactivity^43^. In contrast, H501–018, representative of the predominant RBD-specific competition group, neutralized HCoV-HKU1 less potently but engaged both “up” and “down” RBD conformations, reminiscent of SARS-CoV-2 Class 2 anti-RBD antibodies^33,42^. The distinct epitope targeted by H501–018, which is separate from both TMPRSS2 and H501–008 binding sites, highlights the presence of multiple antigenic surfaces within HCoV-HKU1 RBD domain capable of eliciting neutralizing responses. Together, these findings delineate key neutralization mechanisms and reveal conserved epitopes with potential relevance for vaccine development.

Indeed, identification of neutralizing mAbs (H501–012 and -022) that engage the TMPRSS2 receptor binding motif further highlights this region’s immunological vulnerability. Analogous to SARS-CoV-2 antibodies that block ACE2 engagement and potently neutralize infection^42,44^, our findings suggest a parallel neutralization mechanism in an endemic beta-CoV. Notably, for SARS-CoV-2, antibodies recognizing non-RBD regions also contribute to neutralization, which are critical owing to increased mutation rates in S RBD, rendering RBD-specific Ab responses susceptible to escape through viral evolution and thereby affecting efficacy of RBD targeting mAbs^45–47^. Similarly, identification of diverse mAbs spanning different regions of the HCoV-HKU1 S other than RBD expands this concept to endemic CoVs and supports TMPRSS2 adjacent regions as viable pan-CoV vaccine targets. Although the NTD and S2 directed mAbs isolated in this study did not neutralize HCoV-HKU1 pseudovirus, both NTD and S2 mAbs have been shown to mediate neutralization in other CoVs^48,49^. A caveat here is that our pseudovirus system necessitates TMPRSS2 overexpressing cells, perhaps emphasizing neutralization potential of RBD mAbs. Nonetheless, future structural and functional characterization of HCoV-HKU1 non-RBD mAbs will provide further clarity.

Identification of H501–008 as a HCoV-OC43 cross-reactive mAb highlights existence of conserved antigenic epitopes between different beta-CoVs. Although H501–008 did not efficiently neutralize HCoV-OC43, identification of cross-reactive mAbs has been a major focus of recent CoV research^50,51^. Several reports define broadly neutralizing mAbs isolated from SARS-CoV-2 survivors that cross-react with SARS-CoV and other beta-CoVs^52–55^ whereby mAbs targeting S2 stem helix region were conserved across all known HCoVs^56–59^, and S fusion peptide targeting mAbs are cross-neutralizing across all known HCoVs^60^. These findings emphasize potential for identifying conserved epitopes that can serve as the basis for pan-CoV vaccine development, capable of offering cross-protection against future zoonotic spillovers and seasonal strains alike. Our findings also support using full-length S trimer vaccine antigens, because a vaccine using only S subdomains like RBD will forfeit Ab responses to quaternary epitopes and other non-RBD antigenic sites.

While the primary focus of recent mAb development has centered on epidemic CoVs, this study adds an important perspective by addressing less-appreciated endemic strains. Endemic HCoVs are often viewed as causing less severe infections; however, they persistently cause upper respiratory tract infections, causing hospitalization and life-threatening illnesses in high-risk populations^4–6^ and may influence immune priming for responses to emergent CoVs^3,61^. Lack of licensed vaccines or mAb therapies targeting them reflects both an unfortunate gap in research attention and an opportunity for innovation. Structural characterization provided in this study, including high-resolution mapping of neutralizing and cross-reactive epitopes, offers a strong foundation for rational immunogen design. Strategies such as nanoparticle-based multivalent vaccines could be employed to present these conserved sites to the immune system more effectively. Most notably, the study leveraged pre-pandemic samples, indicating neutralizing and cross-reactive immunity to endemic HCoVs existed before SARS-CoV-2 emergence. This supports the hypothesis that prior HCoV exposure may influence immune responses to SARS-CoV-2, either positively through cross-reactive T or B cell memory or negatively by imprinting suboptimal responses (original antigenic sin).

In conclusion, isolation of mAbs targeting diverse epitopes on the HCoV-HKU1 S, including those with potent neutralizing activity by binding within and around the TMPRSS2 receptor binding site, expands our understanding of the antigenic landscape of endemic HCoVs, but also identifies targets of interest for design of next-generation, broad-spectrum CoV vaccines and therapeutics. Further investigation into similar conserved epitopes across all HCoVs, supported by structural and functional studies, will be essential to improving pandemic preparedness and developing durable protection against evolving CoVs.

## Methods

### Cell Lines

HEK293T (ATCC CRL-3216) cells were maintained in Dulbecco’s modified Eagle’s medium (DMEM) supplemented with 10% FBS, 2 mM glutamine, and 1% penicillin-streptomycin. Huh7 cells were kindly provided by Dr. Mark Heise at University of North Carolina at Chapel Hill and were grown in DMEM supplemented with 10% FBS and anti/anti. Expi293F cells (#A14527, Thermo Fisher Scientific) were maintained in Expi293 expression medium at 37°C, 8% CO_2_, and 70% humidity with shaking at 200 rpm. FreeStyle293-F cells (#R79007, Thermo Fisher Scientific) were maintained in FreeStyle293 expression medium at 37°C, 8% CO_2_, and 70% humidity with shaking at 125 rpm.

### Human PBMCs

Serum and peripheral blood mononuclear cell (PBMC) samples from an individual following a confirmed HCoV-HKU1 infection were collected under the National Institutes of Health, National Institute of Allergy and Infectious Diseases, Vaccine Research Center (NIH, NIAID, VRC) clinical protocol VRC 200 (ClinicalTrials.gov: NCT00067054). All study procedures were reviewed and approved by the Institutional Review Boards (IRB) of the NIH and NIAID, and written informed consent was obtained from all participants before enrollment. Participants received compensation for their time and inconvenience. Before ELISA testing, aliquots of frozen serum samples were heat-inactivated at 56 °C for 1 hour (hr), and assays were conducted in at least two technical replicates. Cryopreserved PBMCs were thawed in a 37°C water bath and immediately transferred to ice-cold staining buffer for downstream processing.

### DNA and protein vector constructs

Full-length S genes from HCoV-HKU1 (GenBank ID: DQ339101), HCoV-OC43 (GenBank: AIL49484.1), SARS-CoV (GenBank ID: AAP13441.1), SARS-CoV-2 (GenBank ID: QHD43416.1), MERS-CoV (GenBank ID: AFS88936.1), and MHV A59 (GenBank ID: AF029248.1) were cloned into mammalian expression vector VRC8400^62,63^ and confirmed by sequencing. S-2P, S1, and RBD were synthesized by PCR using full-length S templates, cloned into plasmid vector VRC8400, and sequence confirmed for protein production. For the expression of HCoV-HKU1 RBD-hFc, RBD residues (326–605) were fused C-terminally with the Fc domain of human immunoglobulin (Ig) previously cloned into a eukaryotic expression vector pCDNA3.1 (GenScript). Proteins used for ELISA binding contained a C-terminal 6x His-tag for protein purification. Genes encoding S ectodomains of HCoV-HKU1 (residues 1–1276), HCoV-OC43 (residues 1–1286), SARS-CoV (residues 1–1190), SARS-CoV-2 (residues 1–1208), and MERS-CoV (residues 1–1291) used to generate S-2P proteins have been described previously^9,10,20^. S-2P constructs used for ELISA binding contained a C-terminal T4 fibritin trimerization motif, an HRV3C protease cleavage site, a Twin StrepTag, and an 8x His-tag for protein purification. Constructs used for generating S-based probes contained an Avi-tag for biotinylation. Proteins were expressed by transfecting vectors into Expi293F cells as described previously^27^. Transfected cell culture supernatants were collected and purified using HisTrap HP Hiload 16/60 Superdex columns (GE Healthcare).

### Mammalian protein expression and purification

Mammalian expression plasmids encoding HCoV-HKU1 RBD (residues 310–606 of isolate N5), HCoV-OC43 S1-CTD (residues 318–608), HCoV-HKU1 NTD (residues 1–294 of isolate N5), HCoV-HKU1 S (residues 1–1276 of isolate N5), HCoV-OC43 S (residues 1–1286), the heavy chain of H501–008 with an HRV3C cleavage site in the hinge between the CH1 and the CH2, the light chain of H501–008, the heavy chain of H501–101 with an HRV3C cleavage site in the hinge between the CH1 and the CH2 and the light chain of H501–101 were transfected into FreeStyle 293-F cells using polyethylenimine (PEI). Proteins containing a C-terminal HRV3C cleavage site, an Fc-tag, and an 8X HisTag (HCoV-HKU1 RBD, HCoV-OC43 S1-CTD, HCoV-HKU1 NTD) and IgGs (H501–008 and H501–101) were purified using Protein A resin (Pierce Biotechnology). HCoV-HKU1 S-2P protein, containing a C-terminal HRV3C cleavage site, a T4 fibritin trimerization motif, an 8xHisTag and a Twin-Strep-Tag, was purified using StrepTactin resin (IBA Lifesciences). Affinity-purified HCoV-HKU1 RBD, HCoV-OC43 S1-CTD, HCoV-HKU1 NTD, H501–008 IgG and H501–101 IgG were purified by size-exclusion chromatography using a Superdex 200 Increase column (GE Healthcare) in 2 mM Tris pH 8.0, 200 mM NaCl, 0.02% NaN3. Affinity-purified HCoV-HKU1 and -OC43 S-2P were purified by size-exclusion chromatography using a Superose 6 column (GE Healthcare) in 2 mM Tris pH 8.0, 200 mM NaCl, 0.02% NaN3. H501–08 and H501–101 IgGs were digested by cleavage with 5% (wt/wt) HRV3C protease on ice for 4 hrs. Cleaved protein was passed over Protein A resin and NiNTA resin (Thermo Fisher Scientific) to remove cleaved Fc and excess protease. H501–008 and -101 Fabs were then purified by size-exclusion chromatography using a Superdex 200 Increase column in 2 mM Tris pH 8.0, 200 mM NaCl, 0.02% NaN3.

### Isolation of mAbs by single B cell sorting

Cryopreserved human PBMCs were thawed and stained with Live/DEAD Fixable Violet Dead Cell Stain (Life Technologies) for single B cell sorting. After washing, cells were stained with a cocktail of anti-human antibodies, including CD3 (clone SP34–2, BD Biosciences), CD4 (clone OKT4, BioLegend), CD8 (clone RPA-T8, BioLegend), CD14 (clone M5E2, BioLegend), CD20 (clone 2H7, BioLegend), IgG (G18–145, BD Biosciences) and subsequently stained with fluorescently labeled HCoV-HKU1 S-2P (APC), SARS-CoV S-2P (BV786) and MERS-CoV S-2P (PE) probes (Extended Data Fig 2). Probe-positive single B cells were sorted into 96-well plates containing lysis solution as previously described^64–66^. Ig heavy and light chain mRNAs were reverse-transcribed and amplified by nested PCR using published primers^67^. Paired heavy and light chain cDNA sequences were cloned into expression vectors containing constant regions of human Ig heavy (γ) and light (κ or λ) chains. IgG was expressed by co-transfecting Expi-293 cells with equal amounts of paired heavy and light chain plasmids, followed by antibody purification using Protein A Fast Flow (GE Healthcare) according to manufacturer’s instructions. Antibody heavy and light chain sequences were compared to human Ig germline sequences using IMGT/V-QUEST^68,69^.

### Enzyme-linked immunosorbent assay (ELISA)

96-well Nunc MaxiSorp plates (Thermo Fisher Scientific) were coated with recombinant S-2P (HCoV-HKU1, HCoV-OC43, SARS-CoV or MERS CoV), HCoV-HKU1 S1, or HCoV-HKU1 RBD proteins at 1 μg ml-1 in 1X PBS at 4°C overnight. Following 90-minute-blocking with PBS-Tween + 5% non-fat dairy milk, plates were either incubated with serial dilutions of mAbs or heat-inactivated human sera samples (1:200, 4-fold, 8x) for an hr. Anti-human IgG horseradish peroxidase (HRP) conjugated secondary Abs (Jackson Laboratory) were used. To detect binding IgG, plates were developed with 3,3’,5,5’-tetramethylbenzidine (TMB) (KPL) and stopped by 1 N sulfuric acid. Readouts of OD450 were collected using SpectraMax Paradigm (Molecular Device). Endpoint titers were calculated at 4x background of secondary alone wells.

### Competition binding analysis by ELISA

mAb antibody mapping was performed using competition ELISA, using previously published methods^64,70^. Briefly, mAbs were biotinylated using the EZ-Link Sulfo-NHS-Biotinylation Kit (Thermo Fisher Scientific) and titrated on HCoV-HKU1 S-2P coated plates. Avidin D-HRP conjugate (Vector Laboratories) and TMB (KPL) were used for color development. Absorbance at 450nm (OD450) was measured using a SpectraMax Plus plate reader (Molecular Devices). The concentration of biotinylated mAb within the linear range of the titration curve was chosen for the competition ELISA. Unlabeled competitor mAbs were serially diluted and added to S-2P-coated plates. After a 30 min incubation at room temperature, biotinylated mAbs were added, and OD readings were recorded. Biotinylated mAb alone served as a binding control. Percent inhibition of binding was calculated as follows: % competition = [1- (OD with competing mAb)/OD without competing mAb)] ×100.

### Competition binding analysis by biolayer interferometry (BLI)

mAb epitope mapping was also performed using biolayer interferometry, as previously described^71^, on the Octet HTX instrument (ForteBio). Briefly, his-tagged HCoV-HKU1 S-2P was loaded onto hydrated Anti-Penta-HIS (HIS1K) biosensors (ForteBio) for 5 minutes (min). The sensors were then incubated in blocking buffer (1% BSA, 0.01% Tween-20, 0.02% Na N_3_ in PBS) for 1 min prior to incubation with competitor mAbs for 5 min. After binding of the competitor mAbs, sensors were incubated in blocking buffer for 1 min, followed by incubation with analyte mAbs for 5 mins. Percent competition was calculated using the following equation: % competition = [1- (Analyte binding in the presence of competitor mAb) / (Analyte binding in the absence of competitor mAb)] × 100.

### mAb affinity measurements by BLI

Anti-hIgG Fc capture sensortips (FortéBio) were soaked in running buffer composed of 10 mM HEPES pH 7.5, 150 mM NaCl, 3 mM EDTA, 0.005% Tween 20 and 1 mg/mL BSA for 20 min before being used to capture mAbs H501–003, -007, -008, -009, -010, -012, -013, -014, -015, -016, -018, -020 or -022 to a level of ~1.4 nm in an Octet RED96 (FortéBio). Tips were then dipped in serial dilutions of either 50–1.5625 nM HCoV-HKU1 NTD (for H501–007 and -010), 10–0.3125 nM HCoV-HKU1 RBD (for H501–003, -008, -009, -012, -013, -014, -015, -016, H501–018, -020, and -022), or 200–3.125 nM HCoV-HKU1 S-2P (for H501–101) before being dipped into wells containing only running buffer to measure dissociation. Data were reference-subtracted in Octet Data Analysis software v11.1 (FortéBio) and fit to a 1:1 binding model.

### mAb affinity measurements by surface plasmon resonance (SPR)

Affinity-purified Fc- and His-tagged HCoV-HKU1 RBD or HCoV-OC43 S1-CTD was immobilized to a single flow cell of an NTA sensor chip at a level of ~300 response units (RUs) per cycle using a Biacore X100 (GE Healthcare). The sensor chip was doubly regenerated using 0.35 M EDTA and 0.1 M NaOH followed by regeneration with 0.5 mM NiCl2. Three samples containing only HBS-P pH 8.0 running buffer were injected over both ligand and reference flow cells, followed by H501–008 Fab serially diluted from 25–0.78125 nM, with a replicate of the 6.25 nM concentration. The resulting data were double-reference subtracted and fit to a 1:1 binding model using the Biacore X100 Evaluation software. Twin-Strep-tagged and His-tagged HCoV-HKU1 S or HCoV-OC43 S was immobilized to a single flow cell of an NTA sensor chip at a level of ~250 RUs per cycle and H501–101 Fab binding was measured over a serial dilution from 25–0.78125 nM with a replicate of the 6.25 nM concentration. Sensor chip regeneration, reference subtraction, and data processing were performed under the same conditions described above.

### HCoV-HKU1 pseudovirus neutralization assay

Pseudotyped HCoV-HKU1 coronavirus was generated as described elsewhere with minor modifications^38,72^. Briefly, full-length HCoV-HKU1 S gene (GenBank accession number-DQ339101) or HCoV-HKU1 S with indicated amino acid substitutions lacking the native signal peptide and endoplasmic retention signal sequence was cloned into the pCDNA 3.1 expression vector with a tissue plasminogen activator signal sequence (MDAMKRGLCCVLLLCGAVFVSA). 10 μg of plasmid was transfected into HEK293T cells using PEI (Polysciences) at a 1:3 ratio in OPTI-Minimum Essential Media (Opti-MEM, Gibco, ThermoFisher Scientific). Twenty-four hrs post-transfection, cells were infected with VSVΔG/Luc virus and incubated for 2 h at 37°C with 5% CO_2_. The cells were washed thrice with PBS and complete DMEM (DMEM with 10% FBS and 1% penicillin-streptomycin), with anti-VSV-G antibody (I1-mouse hybridoma supernatant diluted to 1:25 from ATCC- CRL-2700) was added to the cells to reduce parental virus background and incubated for 24 hrs. Cell supernatants were harvested 24 hrs post-infection, cell debris was removed by centrifugation at 3000 rpm for 10 min, filtered (0.45μm), and aliquots were stored at −80°C until used. For pseudovirus titration, HEK293-TMPRSS2 cells grown in 96-well black and white plates were infected with 50 μL of serially diluted virus stock (2-fold dilutions). After 24 hr incubation at 37 °C in a CO_2_ incubator, cells were lysed using cell lysis buffer (Promega) following the manufacturer’s instructions, and subsequently, 50 μL of luciferase substrate (Promega) was added to the cells. RLU values were calculated and plotted against log10 virus dilutions in GraphPad Prism.

For pseudovirus neutralization assays, HEK293T cells at 80% confluency in 100mm plates were transfected with 10 μg of TMPRSS2 plasmid and incubated for 6 hr. Transfected cells (HEK293T-TMPRSS2) were trypsinized, counted, and reseeded at ~20,000 cells/well in 96-well black and white plates (Revvity) and incubated at 37°C with 5% CO_2_ overnight. The following day, mAb serial dilutions (4-fold, 8x, starting at 20 μg/mL) were prepared in DMEM (Gibco, ThermoFisher Scientific) in 90 μL final volume. 90 μL of HCoV-HKU1 pseudovirus (100,000 RLU) was added to each well, and the pseudovirus/Ab mixture was incubated at 37°C for 45 min. 50 μL of pseudovirus/Ab mixture was added to HEK293T-TMPRSS2 cells and incubated for 2 hr at 37°C followed by the addition of 100 μL of DMEM with 10 % FBS and 1 % Penstrep. Twenty hrs post-infection, the cells were lysed using cell lysis buffer (Promega) following the manufacturer’s instructions, and subsequently, 50 μL of luciferase substrate (Promega) was added to the cells. Luciferase readout (RLU) was measured using a Spectramax iD5 plate reader (Molecular Sciences). RLU values in triplicate were plotted and normalized in GraphPad Prism, with a baseline value of pseudovirus alone set to 0% neutralization and uninfected cells representing 100% neutralization. Nonlinear regression was employed to calculate the IC_50_ values from the fitted curves.

### HCoV-OC43 neutralization assay

Huh7 cells were seeded at 20,000 cells/well in a Poly-L-Lysine (Gibco, Thermo Fisher Scientific) coated 96-well plate. The following day, mAbs (H501–008, H501–101, and CDC2-C2^73^) were serially diluted (4-fold, 9x starting at 40 μg/mL) in Optimem (Gibco, ThermoFisher Scientific) containing 2% FBS, NEAA (Gibco), and anti/anti (Thermo Fisher Scientific). Equal volumes of HCoV-OC43 (VR-1558 strain) (MOI=0.025) were added to each well and incubated at 37°C for 1 hr. Following incubation, 50 μl of each virus/antibody series was added to Huh-7 cells in quadruplicates and incubated at 32°C for 1 hr. After 1 hr, cells were overlaid with Optimem containing 1% carboxymethyl cellulose, 2% FBS, NEAA, and anti/anti. After 72 h incubation at 32 °C, cells were fixed with 10% buffered formalin (Thermo Fisher Scientific), permeabilized using 0.1% Triton X-100 (Sigma-Aldrich), and blocked in PBS containing 1% BSA and 0.2% skim milk. Endogenous peroxidase activity was quenched with 3% hydrogen peroxide (Thermo Fisher Scientific). HCoV-OC43 antigen was detected using a mouse anti-OC43 nucleoprotein primary antibody (Millipore MAB9013) followed by an HRP-conjugated goat anti-mouse secondary antibody (KPL 474–1806) and visualized with DAB reagent (Thermo Fisher Scientific). Infected cell foci were imaged and quantified using a CTL ImmunoSpot ELISpot reader. The effective concentration resulting in a 50% reduction in viral replication was defined as EC_50_. EC_50_ values were calculated from a dose-response curve using a 4-parameter (variable slope) equation (Equation (1)) in GraphPad Prism. Y = 100/(1 + 10^((LogEC_50_-X)*HillSlope)) where Y represents % inhibition and X represents Ab concentration. Mock-infected cells were used as a control for 100% inhibition, and cells treated with virus alone were used as the baseline for 0% inhibition.

### Cell surface mAb binding assay

HEK293T cells were transiently transfected with plasmids encoding full-length S proteins from HCoV-HKU1, HCoV-OC43, MERS-CoV, SARS-CoV, MHV-CoV, or HCoV-HKU1 with indicated amino acid substitutions, using Lipofectamine 3000 (Thermo Fisher Scientific) according to the manufacturer’s protocol. After 40 hrs, cells were harvested and incubated with mAbs (1 μg/ml) for 30 min. Following antibody incubation, cells were washed and stained with an allophycocyanin-conjugated anti-human IgG secondary antibody (Jackson ImmunoResearch Laboratories, 709–136-149) for an additional 30 min. Cells were then washed and fixed with 1% paraformaldehyde (Electron Microscopy Sciences). Samples were acquired in a BD LSR Fortessa X-50 flow cytometer (BD Biosciences) and analyzed using FlowJo V.10.10.0 (BD Biosciences).

### S-TMPRSS2 binding assay

To determine whether HCoV-HKU1 mAbs interfere with S binding to TMPRSS2, we performed a S binding assay on HEK293T-TMPRSS2 cells as described elsewhere^74^. Briefly, TMPRSS2 was transiently expressed in HEK293T cells seeded on coverslips (Electron Microscopy Sciences, 72290–02) for immunofluorescence staining or 6 well plates (Costar, 3516) for flow cytometry analysis (FACS). Before flow cytometry analysis, HEK293T-TMPRSS2 cells seeded in 6-well plates were trypsinized and added to round-bottom plates (Costar, 3788). The transfected cells were blocked with 1 % BSA for 30 min at 4°C followed by a PBS (Gibco, Thermo Fisher Scientific) wash. Prior to addition of HCoV-HKU1 RBD with TMPRSS2 expressing cells, recombinant protein was incubated independently with 10 μg/ml of HCoV-HKU1 mAbs and incubated for 30 min at 4 °C. The mAb/CTD mixture was allowed to bind to HEK293T-TMPRSS2 for 1 hr at 4°C, followed by two PBS washes. For immunofluorescence staining of HCoV-HKU1 RBD, binding was detected using anti-human Alexa Fluor 594 (Invitrogen, A11014), whereas for flow cytometry analysis, staining was performed with anti-human FITC (Bethyl, A80–119F). Following secondary Ab staining, cells were washed with PBS, fixed in 4% PFA and processed independently for confocal imaging or FACS. Images were acquired in a Nikon Eclipse T2 confocal microscope and were processed using Nikon Elements V.5.21.0, whereas FACS samples were acquired in iQue 3, Sartorius, and analyzed using FlowJo V.10.10.0.

### X-ray crystallography

HCoV-HKU1 RBD (residues 310–606 of isolate N5) was treated with HRV3C protease, as described above, and the digested protein was passed over Ni-NTA resin to remove cleaved tags and excess protease. Un-tagged HCoV-HKU1 RBD was then mixed with a molar excess of H501–008 Fab and passed over a Superdex 75 using 2 mM Tris pH 8.0, 200 mM NaCl, 0.02% NaN3. The purified complex was then concentrated to 6.89 mg/mL and used to prepare sitting-drop vapor diffusion crystallization trays. Crystals grown in 0.1 M Tris pH 8.5, 0.2 M MgCl2 and 25% PEG 3350 were soaked in mother liquor supplemented with 20% glycerol and frozen in liquid nitrogen. Diffraction data were collected to a resolution of 2.60 Å at the ALS beamline 5.0.1. Diffraction data were indexed and integrated using iMOSFLM^75^ before being scaled in AIMLESS^76^. The HCoV-HKU1 RBD + H501–08 Fab dataset was phased by molecular replacement in Phaser-MR^77^ using coordinates from PDBs 4YDJ, 3TV3, and 5KWB as search ensembles. The resulting molecular replacement solution was iteratively rebuilt and refined using Coot^78^, ISOLDE^79^, and Phenix^80^. Crystallographic software packages were curated by SBGrid^81^.

### CryoEM sample preparation

Fabs were immediately complexed with 6–7 mg/mL HCoV-HKU1 S-2P at a 1.1:1 molar ratio Fab to S protomer followed by the addition of fluorinated octyl-maltoside (FOM, Anatrace) to a final concentration of 0.02% w/v. Complexes (3 μL) were applied to Quantifoil Cu 1.2/1.3 300 mesh grids that were freshly glow-discharged at 0.38 mbar, 15 mA, 60 seconds using a PELCO easiGLOW (Ted Pella). Grids were blotted using a Vitrobot cryo-plunger (Thermo Fisher Scientific) for 3–4 seconds at 6 °C, 100% humidity, prior to plunging into liquid ethane.

### CryoEM data collection and processing

For HCoV-HKU1 S-2P with H501–008 Fab complex, single-particle data were collected on a Glacios 2 Cryo-TEM equipped with a Falcon 4i detector (Thermo Fisher Scientific). The microscope was operated at 200 kV, 150000x magnification, and images were collected as EER frames at 0.923 Å/pixel (unbinned) using EPU automated data collection software. Images were collected in counting mode, one shot per hole, applying beam shift with a total exposure of 40 el/Å^2^ and defocus values ranged from 0.7 – 2.5 μm. For the HCoV-HKU1 S-2P with H501–018 Fab and HCoV-HKU1 S-2P with H501–022 Fab complexes, single-particle data were collected on a Titan Krios G2 Cryo-TEM (Thermo Fisher Scientific). The microscope was operated at 300 kV, 130000x magnification, and images were collected as EER frames at 0.92 Å/pixel (unbinned) using EPU automated data collection. Images were collected in counting mode, three shots per hole, applying beam shift with a total exposure of 30 el/Å^2^ and defocus values ranging from 0.4 – 2.5 μm. Additional data collection parameters can be found in Supplementary Table 2.

For all cryoEM datasets, patch motion corrected, patch CTF estimation, and reference-free particle picking using circular picks ranging from 100–300 Å were carried out in cryoSPARC v4.6^34,82^. Particles were extracted using a box size of 480 pixels (HCoV-HKU1 S-2P with H501–018 or H501–022) or 500 pixels (HCoV-HKU1 S-2P with H501–008) and downsampled 4x prior to 2D classification. Particles selected from 2D classification were inputted for Ab-Initio generation assigning 2–4 classes, and heterogeneous refinement to obtain a starting cryoEM map of S-Fab complexes for downstream homogeneous refinements, local refinements and global non-uniform refinements. HCoV-HKU1 S-2P with H501–022 Fab was further 3D classified into 4 classes in Relion v5 while applying Blush Regularization^83,84^. To separate out the different states of HCoV-HKU1 S-2P with H501–018 Fab, particles were taken into Relion v5, and local masked 3D classification with Blush Regularization was performed, assigning 3 classes^83,84^. The circular mask was generated from apoferritin (EMD-20026, low-pass filtered to 15 Å, extending by 3 pixels with a soft-edge of 6 pixels) resampled onto one protomer of the S-Fab density to sort out particles with Fabs bound to either 2-up 1-down RBD or 2-down 1-up RBD^85^. Particles classified in Relion were imported back into cryoSPARC for another round of nonuniform refinement. Resolutions of all maps were generated applying the Fourier Shell Correlation (FSC) cutoff of 0.143 criterion in cryoSPARC. Data processing statistics and workflow summary can be found in Supplementary Table 2 and Extended Data Fig. 6, respectively.

### CryoEM structure modeling, refinement, and analyses

Coordinates for initial models were fit into cryoEM map densities using ChimeraX prior to real space refinements and validations^86–88^. PDB codes used for modeling HCoV-HKU1 S were 8Y8A, 8Y8H, 8Y8G for S with 3 RBD up, 2-up-1-down RBD, and 2-down-1-up RBD, respectively^39^. For S structures, rigid body docking was performed in ChimeraX prior to validation in Phenix^86–89^. *N*-linked glycans were modelled where there was density for glycans near the N-X-T/S S sequences. Proline residue substitutions at positions 902, 980, and 1023 were mutated back to the original sequences followed by Real Space Refinements in Coot^90^. Starting models of variable heavy (V_H_) and variable light (V_L_) chains were obtained from the IMGT 3D structure database^91^. PDB codes 7T3M and 5O7P^92,93^, respectively, were docked into the V_H_ or V_L_ density of the local refined map for H-022 Fab bound to S 2P. PDB codes 4FQK and 4QHK^94,95^, respectively, were docked into the local refined map for H-018 Fab bound to S 2P. Model building and real space refinements for Fab-RBD structures were carried out in Coot and Phenix ^89,90^. BSA analyses were carried out using PDBePISA^35^. Total BSA was determined by taking the sum of all residue BSA contributed. Interacting residues were identified by using a 4-Å search cutoff in Coot and ChimeraX^86–88,90^.

### mAb footprint analysis

HCoV-HKU1 S sequences from genotypes A, B, and C were retrieved from National Center for Biotechnology Information (NCBI), GenBank, and multiple sequence alignment was performed in MEGA11^96^, using the MUSCLE algorithm. Aligned sequences were trimmed to represent RBD regions, 441–564, and mAb binding residues were highlighted as red (H501–008), purple (H501–018), and green (H501–022).

### Data Availability

Source data supporting the findings of this study are available within this paper and its supplementary files. Source data has also been deposited to Figshare and can be accessed here: 10.6084/m9.figshare.30468065. Additional source data inquiries should be directed to the corresponding author, Kizzmekia Corbett-Helaire (kizzmekia_corbett@hsph.harvard.edu). The atomic models and cryo-EM maps generated for H501–018 bound to HCoV-HKU1 S-2P and H501–022 bound to HCoV-HKU1 S-2P have been deposited to the Protein Data Bank (PDB; http://www.rcsb.org/) and the Electron Microscopy Databank (EMDB; http://www.emdataresource.org/) under accession codes PDB: 9YGN, 9YGO, 9YGP, 9YGQ, and 9YGR and EMD-72935, EMD-72936, EMD-72937, EMD-72938, and EMD-72939, respectively. Additionally, the atomic coordinates for H501–008 bound to RBD are deposited to the Protein Data Bank under accession code 9YXW, and the cryo-EM map for H501–008 bound to HCoV-HKU1 S-2P is deposited to the Electron Microscopy Databank under accession code EMD-72934.

### Material and Resource Availability

Requests for reagents and resources must be directed to the corresponding author, Kizzmekia Corbett-Helaire (kizzmekia_corbett@hsph.harvard.edu), and will be provided upon execution of a Material Transfer Agreement. If the material was obtained under use restriction, the inquiry will be forwarded to appropriate party.

## Supplementary Material

Supplement 1

Supplement 2

## Figures and Tables

**Table 1.** HCoV-HKU1 mAb binding specificity, affinity, and neutralization potency.

Specificity	Name	ELISA EC50 (μg/mL)^1^			Octet^2^			Neutralization(μg/mL)^3^	
		S-2P	S1	RBD	K_D_ (M)	K_a_ (M^−1^s^−1^)	K_d_ (s^−1^)	IC50	IC80
RBD^4^	H501-003	0.0122	0.0277	0.0176	5.08E-10	2.19E+05	1.11E-04	0.57	1.59
	H501-008	0.0055	0.0049	0.0068	9.37E-10	3.36E+05	3.15E-04	0.05	0.12
	H501-009	0.0041	0.0134	0.0095	5.44E-09	7.03E+04	3.83E-04	4.91	>10
	H501-012	0.0059	0.0079	0.0097	9.69E-10	3.12E+05	3.02E-04	0.02	0.06
	H501-013	0.0077	0.0144	0.0155	9.15E-10	2.28E+05	2.09E-04	3.77	>10
	H501-014	0.0084	0.0177	0.0188	2.65E-10	1.92E+05	5.10E-05	1.66	3.75
	H501-015	0.0041	0.0094	0.0098	5.68E-10	2.06E+05	1.17E-04	1.32	2.43
	H501-016	0.0054	0.0078	0.0079	1.76E-10	1.85E+05	3.26E-05	2.54	6.09
	H501-018	0.0191	0.0574	0.0306	3.70E-10	2.83E+05	1.05E-04	0.28	0.74
	H501-020	0.0094	0.0193	0.0172	<1.0E-12	3.33E+05	<1.0E-07	0.32	0.85
	H501-022	0.0064	0.0144	0.0160	2.01E-09	2.76E+05	5.55E-04	0.01	0.04
NTD^5^	H501-007	0.0327	0.3471	-	2.15E-07	2.34E+04	5.04E-03	>10	>10
	H501-010	0.0076	0.0177	-	5.88E-09	4.78E+05	2.81E-03	>10	>10
S2^6^	H501-101	0.0060	-	-	<1.00E-12	1.85E+05	<1.0E-07	>10	>10

1 Binding of mAbs was assessed by ELISA to HCoV-HKU1 S-2P, S1, and RBD proteins. EC50 was calculated. “-” = no curve generated.

2 Affinity of mAbs bound to proteins, per Extended Data Fig. 3, was measured using biolayer interferometry and K_D_, Ka and Kd were calculated.

3 Neutralization activity was measured using a HCoV-HKU1 neutralization assay. IC values represent geometric mean titers (GMT) from two independent experiments.

4 mAbs were determined to be RBD specific if they bound to all three proteins.

5 mAbs were determined to be NTD specific if they bound to S-2P and S1.

6 mAbs were determined to be S2-specific if they only bound S-2P.
