# Supplementary material for: Human Coronavirus HKU1 Neutralizing Monoclonal Antibodies Target Diverse Epitopes Within and Around the TMPRSS2 Receptor Binding Site
Source: bioRxiv. 2025 Oct 30:2025.10.29.685445. Preprint. [Version 1] doi: 10.1101/2025.10.29.685445 (PMC12636560; doi:10.1101/2025.10.29.685445)
Supplement: Supplement 1 [file media-1.pdf]

Human Coronavirus HKU1 Neutralizing Monoclonal Antibodies Target Diverse Epitopes Within and Around the TMPRSS2 Receptor Binding Site

Lingshu Wang[1,16], Jeswin Joseph[2,16], Sheena Vasquez[3,16], Daniel Wrapp[4,11], Timothy P. Sheahan[5], Christian K.O. Dzuvor[2], Osnat Rosen[1,12], Robert N. Kirchdoerfer[6,13], Olubukola M. Abiona[1], Catherine Hammond[7], Wei Shi[1], Sydney P. Moak[7], Wing-Pui Kong[1], Yi Zhang[1], Michael R. Eso[3], Ariane J. Brown[5], Andrew B. Ward[6], Ralph Baric[5], Jason S. McLellan[4], Theodore C. Pierson[1], John Mascola[1,14], Barney S. Graham[1,15], Hadi M. Yassine[8], Christopher O. Barnes[3,9,10,17], Kizzmekia S. Corbett-Helaire[1,2,7,17,18]

[1]Vaccine Research Center; National Institutes of Allergy and Infectious Diseases; National Institutes of Health; Bethesda, Maryland, 20892; United States of America

[2]Department of Immunology and Infectious Diseases; Harvard T.H. Chan School of Public Health; Boston, Massachusetts, 02115; United States of America

[3]Department of Biology; Stanford University; Stanford, CA, 94305; United States of America

[4]Department of Molecular Biosciences; University of Texas at Austin; Austin, Texas, 78712; United States of America

[5]Department of Epidemiology; University of North Carolina at Chapel Hill; Chapel Hill, North Carolina, 27599; United States of America

[6]Department of Integrative Structural and Computational Biology, The Scripps Research Institute, La Jolla, CA,92037; United States of America

[7]Howard Hughes Medical Institute; Chevy Chase, Maryland, 20815; United States of America

[8]Biomedical Research Center, Member of QU Health, Qatar University; Doha, Qatar

[9]ChEM-H Institute, Stanford University; Stanford, 94305, United States of America

[10]Chan Zuckerberg Biohub; San Francisco, 94158, United States of America

[11]Current affiliation: Duke Human Vaccine Institute, Duke University School of Medicine, Durham, NC 27710; United States of America

[12]Current affiliation: Department of Biotechnology, Israel Institute for Biological Research, Ness-Ziona, Israel

[13]Current affiliation: Department of Biochemistry, Institute for Molecular Virology, Center for Quantitative Cell Imaging, University of Wisconsin-Madison, Madison, Wisconsin, 53706; United States of America

[14]Current affiliation: Modex Therapeutics, Weston, MA, 02493

[15]Current affiliation: Medicine and Microbiology, Biochemistry, & Immunology, Morehouse School of Medicine, Atlanta, GA, 30310

[16]Authors contributed equally to this study.

[17]Authors contributed equally to this study.

[18]Correspondance: kizzmekia_corbett@hsph.harvard.edu

Extended Figures

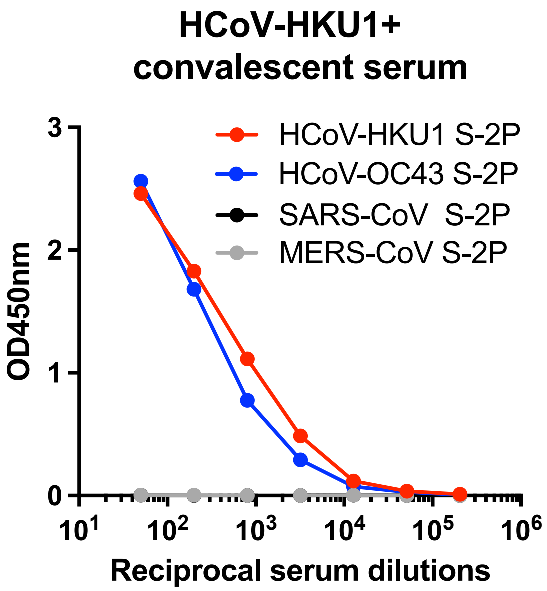

Extended Data Fig. 1. HCoV-HKU1-positive convalescent human serum binds to HCoV-HKU1 and -OC43 prefusion spike (S-2P proteins. Human serum collected 41 days after confirmed HCoV-HKU1 infection was assessed for binding to HCoV-HKU1 (red), HCoV-OC43 (blue), SARS-CoV (black), and MERS-CoV (gray) S-2P by ELISA. SARS-CoV and MERS-CoV datapoints are overlaid on the X-axis.

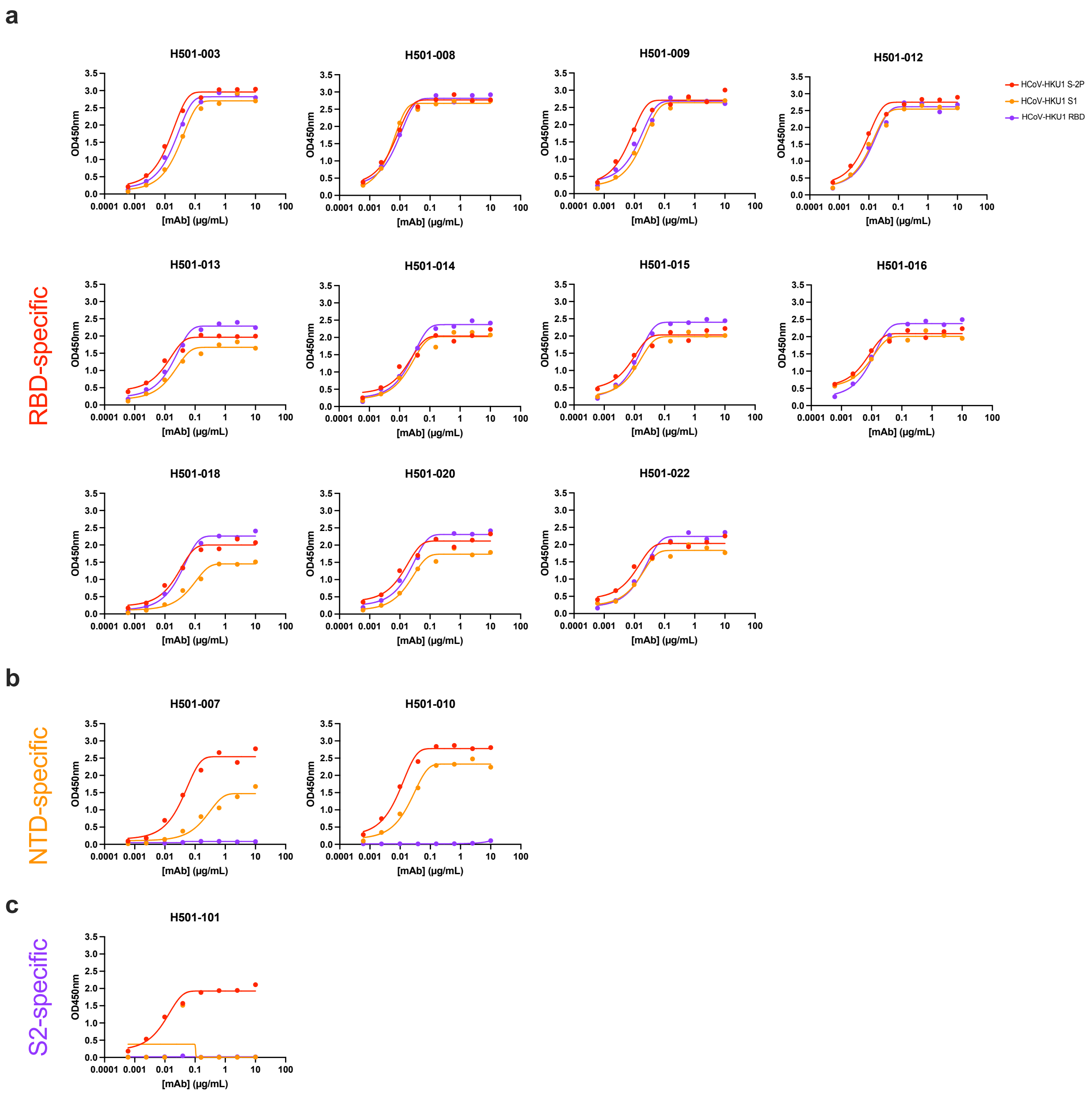

Extended Data Fig. 2. Binding of HCoV-HKU1 mAbs to HCoV-HKU1 S protein subunits. Binding of HCoV-HKU1 mAbs to HCoV-HKU1 S-2P (red), S1 (orange), and S2 (purple) by ELISA. mAbs were determined to be RBD-specific if they bound to all three proteins. mAbs were determined to be NTD-specific if they bound to S-2P and S1. mAbs were determined to be S2-specific if they only bound S-2P.

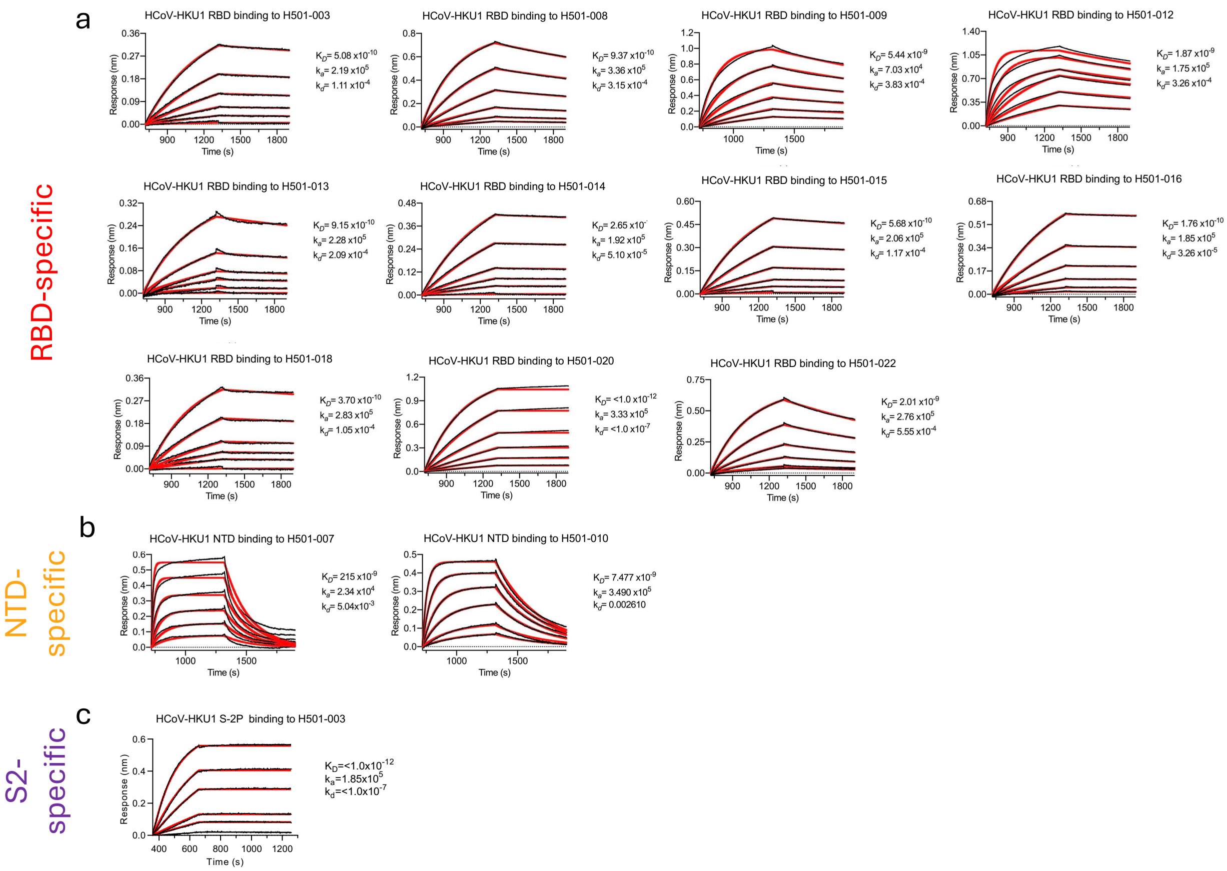

Extended Data Fig. 3. Binding affinity of HCoV-HKU1 mAbs. Binding affinities of RBD (a), NTD (b), and S2-specific (c) mAbs bound to HCoV-HKU1 RBD, NTD, and S-2P, respectively. mAbs were immobilized to biosensors for 1:1 binding kinetic analysis and measured by biolayer interferometry. KD (M), Ka ($M^{-1}s^{-1}$), and Kd (s^{-1}) were calculated **(Table 1)**.

Ab1	Ab2												
	H501-018	H501-013	H501-003	H501-014	H501-015	H501-016	H501-020	H501-009	H501-012	H501-022	H501-008	H501-007	H501-010
H501-018	94	96	105	97	95	91	92	95	49	49	38	19	11
H501-013	96	97	100	100	104	91	97	101	38	46	33	11	15
H501-003	82	82	83	80	78	77	77	84	26	29	27	9	-13
H501-014	95	88	91	90	87	79	88	92	24	34	24	7	-7
H501-015	93	98	110	100	93	104	95	99	46	46	30	18	-1
H501-016	96	99	135	99	103	92	95	101	36	29	11	8	-7
H501-020	97	98	101	98	102	92	97	97	44	43	28	13	-4
H501-009	93	96	103	96	92	89	94	95	27	16	16	-1	-11
H501-012	18	27	29	17	18	24	22	28	90	88	6	21	-3
H501-022	34	23	1	19	14	8	11	18	95	89	18	18	0
H501-008	22	19	-11	0	7	3	13	2	-17	-9	94	15	-10
H501-007	27	33	36	26	29	19	26	33	14	28	43	93	42
H501-010	30	35	42	18	27	16	35	25	26	32	24	43	100

% Inhibition >90% 80-89.99% 60-79.99% 50-59.99% <50%

Extended Data Fig. 4. HCoV-HKU1 mAb competition map. HCoV-HKU1 RBD- (red) and NTD-specific (orange) mAbs were assessed for competition binding to HCoV-HKU1 S-2P bound to mAbs listed in the leftmost column. Analyses were completed by biolayer interferometry (BLI). Percent inhibition of analyte mAb binding by competitor mAb is indicated by color. Dark red = >90% competition, red = 80-99.99% competition, yellow = 60-79.99% competition, and uncolored = <50% competition.

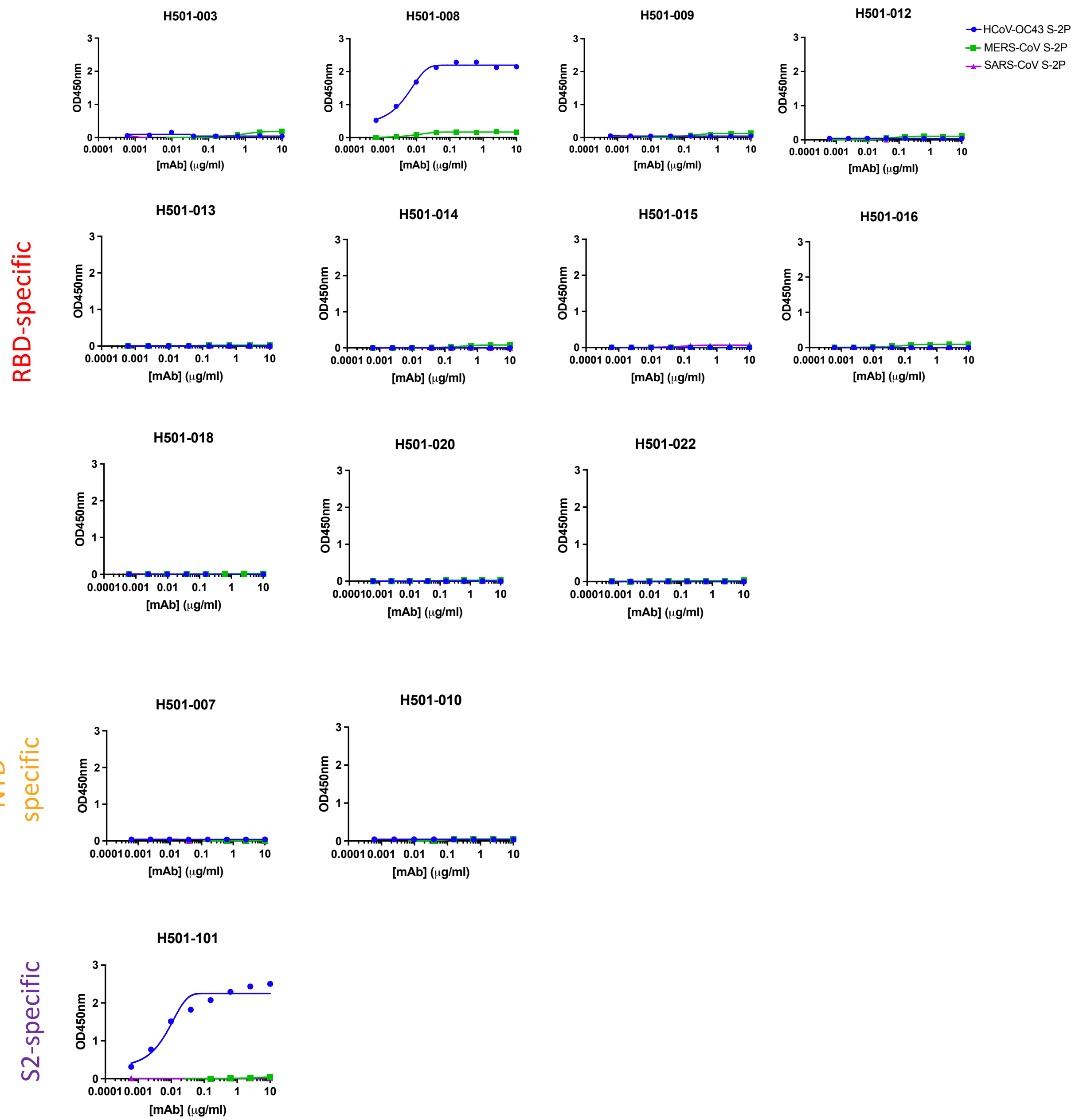

Extended Data Fig. 5. Binding of HCoV-HKU1 mAbs to beta-CoV S proteins. Binding of HCoV-HKU1 mAbs to HCoV-OC43 (blue), MERS-CoV (green), and SARS-CoV S-2P (purple) by ELISA. For all mAbs, with exception of H501-008 and -101, all datapoints are overlaid on the X-axis, indicative of no binding. For H501-008 and -101, MERS- and SARS-CoV S-2P data points are overlaid on the X-axis, indicative of no binding.

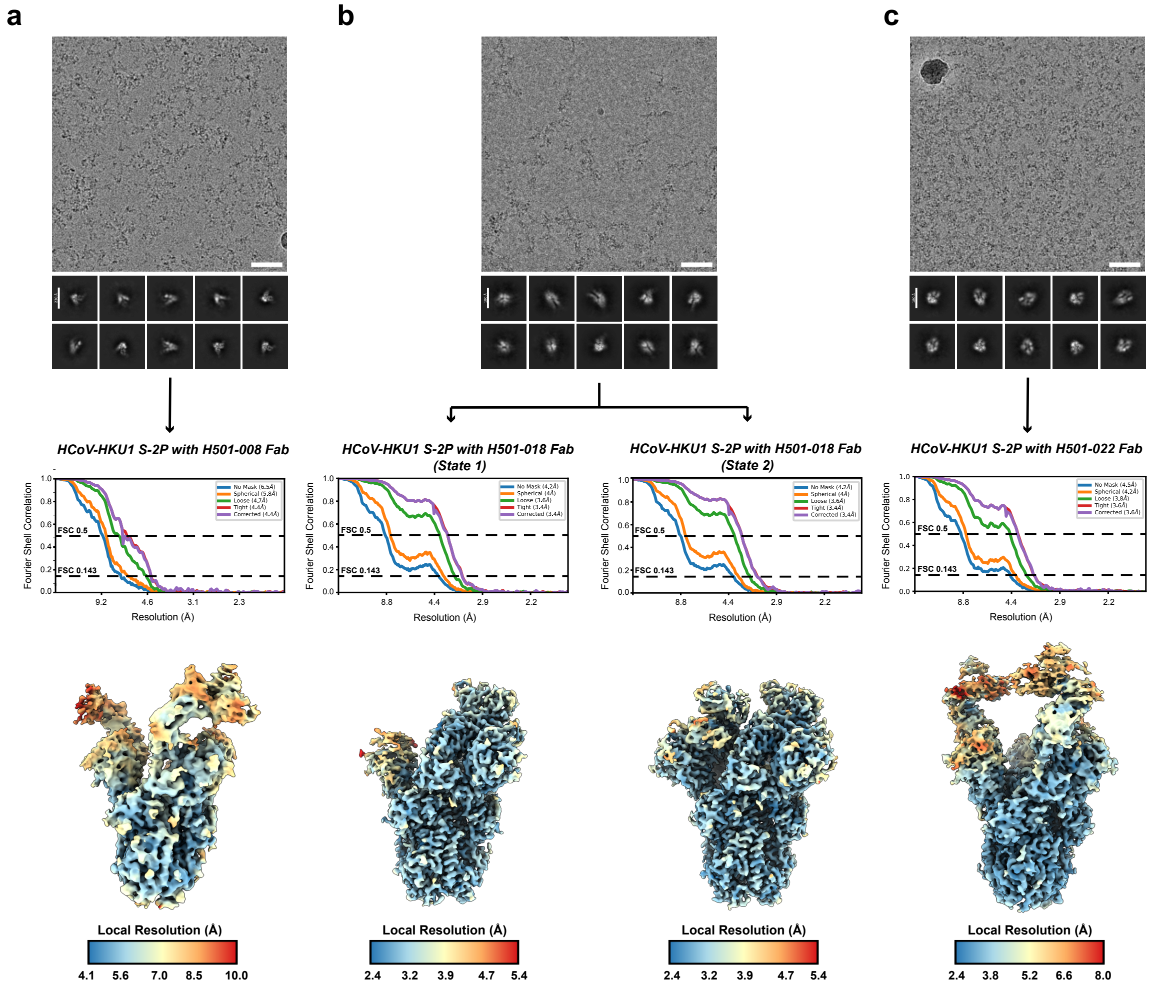

Extended Data Fig. 6. Cryo-EM data processing summary of H501-008, H501-018, and H501-022 complexes. (a-c) Representative micrographs, 2D class averages, Fourier Shell Correlation (FSC) plots, and final map reconstructions of H501-008 Fab – S-2P (a), H501-018 Fab – S-2P (b), and H501-022 Fab – S-2P (c) complexes. Two states were observed for H501-018 Fab complexed to S-2P (b); one state where there are two S1 RBDs facing up and one RBD down (State 1), and another state with two RBDs facing down and one RBD up (State 2). Scalebars (white) for representative micrographs are 50 nm.

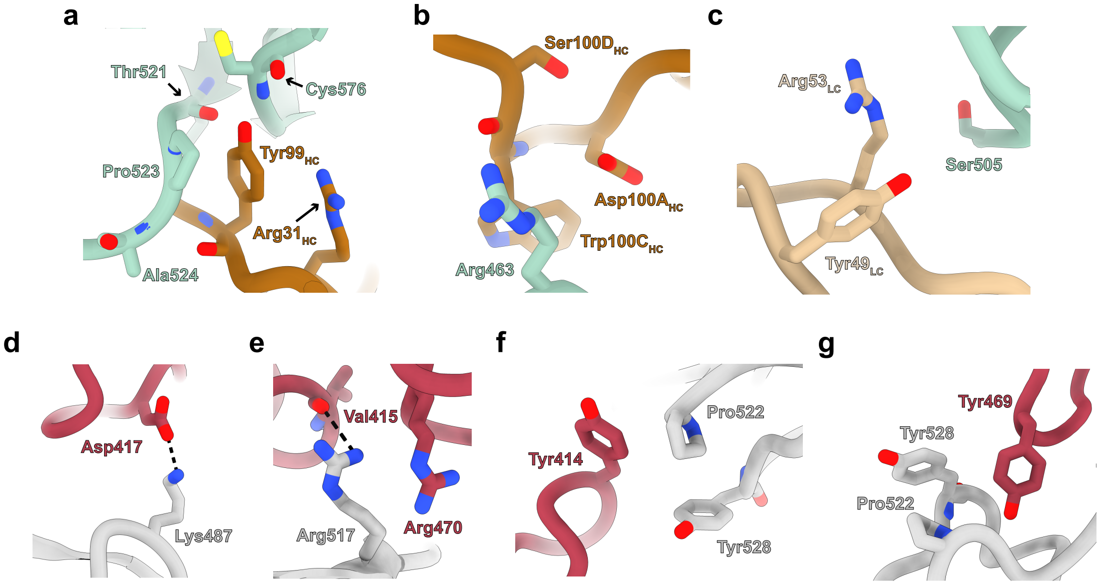

h

	Receptor Binding Domain																												
**HKU1 S Residue Number**	**442**	**443**	**444**	**445**	**446**	**447**	**448**	**449**	**477**	**478**	**479**	**480**	**481**	**482**	**493**	**494**	**495**	**496**	**497**	**498**	**499**	**500**	**501**	**502**	**563**	**564**	**565**	**566**	**567**
**HKU1 S amino acid**	S	S	W	N	R	R	Y	G	A	D	P	S	V	V	A	I	C	P	A	G	T	K	Y	R	S	S	C	F	C
**OC43 S amino acid**	S	T	W	N	K	R	F	G	K	L	N	S	S	-	G	T	C	P	A	G	T	N	Y	L	N	P	C	T	C
**Buried Surface Area (Å^2)**	0	20.2	0	0	20.3	114.5	0.2	0	0	20.6	8.7	69	9	0	0	53.2	18.5	41.9	93.5	36.4	0	0	15.4	0	36.5	45.5	6.6	0	0

Extended Data Fig. 7. Comparisons of HCoV-OC43 S and TMPRSS2 with HCoV-HKU1 S. (a-c) Conserved residues from HCoV-OC43 S1 C-terminal domain (S1-CTD) (PDB 7SB3) are docked near H501-008 by superimposing HCoV-OC43 S1-CTD onto the crystal structure of the HCoV-HKU1 RBD – H501-008 complex. (d-g) HCoV-HKU1 RBD residues that interact with H501-022 (Fig. 5 f-j) are shown interacting with TMPRSS2 (PDB 8Y8B). Colors are as follows: OC43 S1-CTD (light teal), H501-08 V_H (orange), H501-08 V_L (tan), HCoV-HKU1 RBD (light gray), TMPRSS2 (red). (h) Sequence alignment of HCoV-OC43 S (GenBank AIL49484) and HCoV-HKU1 S (GenBank DQ339101). Buried Surface Area (BSA) between HCoV-HKU1 S1-CTD and H501-008 is color coded based on relative BSA values.

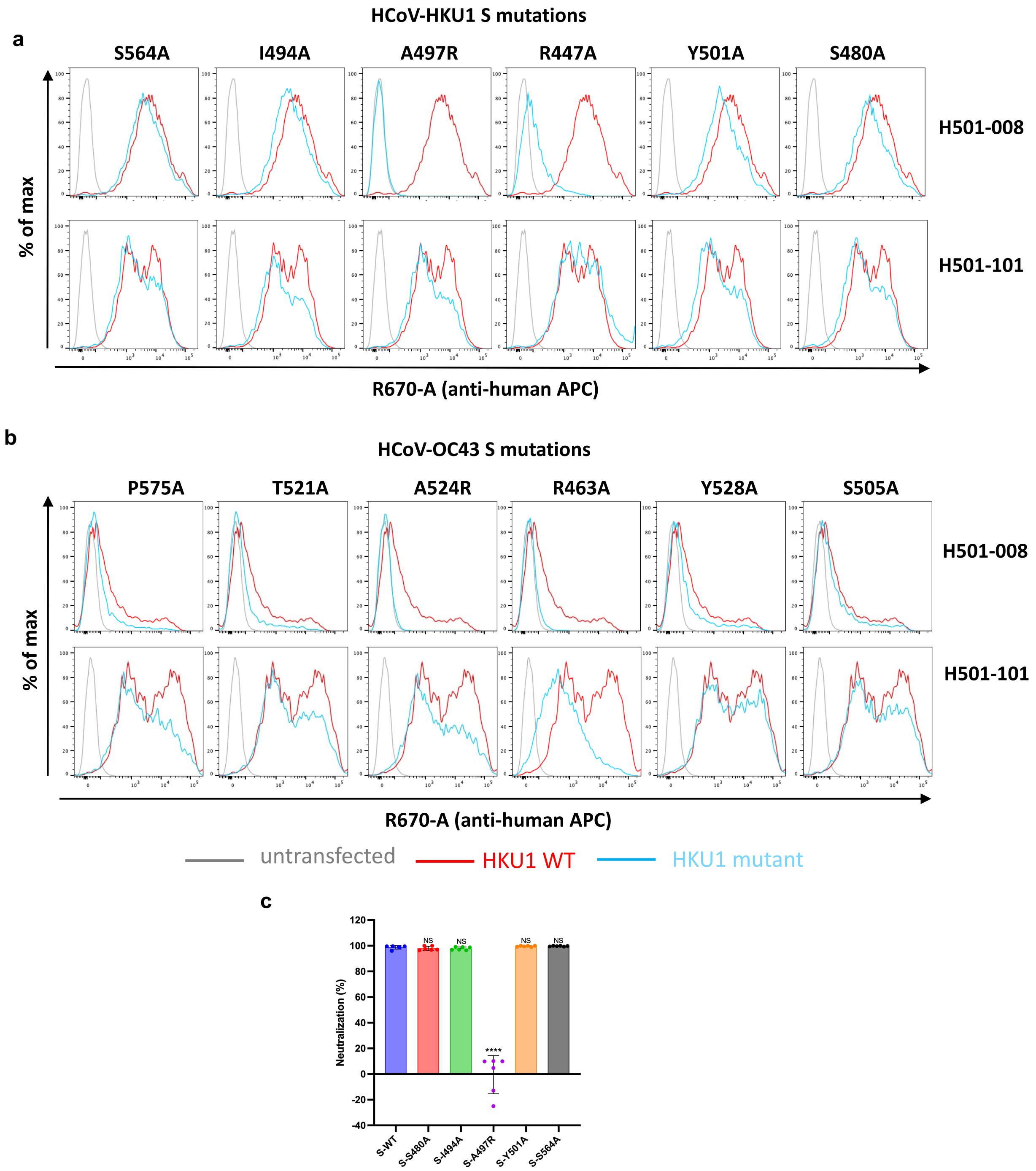

Extended Data Fig. 8. HCoV-HKU1/OC43 RBD-specific mAb H501-008 binding residues. (a-b) H501-008 and -101 were assessed for binding to cell surface-expressed HCoV-HKU1 (a) or HCoV-OC43 (b) S with indicated mutations, as revealed by crystal structure in **Fig. 3**. Percentage of maximum binding to untransfected cells (gray), wild-type S-transfected cells (red), mutant S-

transfected cells (light blue) are shown. (c) H501-008 (10 μg/mL) neutralization of HCoV-HKU1 pseudoviruses with indicated S mutations as revealed by crystal structure in **Fig. 3**. Data shown are mean ± s.d. of n = 2 independent experiments performed in technical triplicates. Statistical analysis was performed using one-way ANOVA; ****P <0.0001.

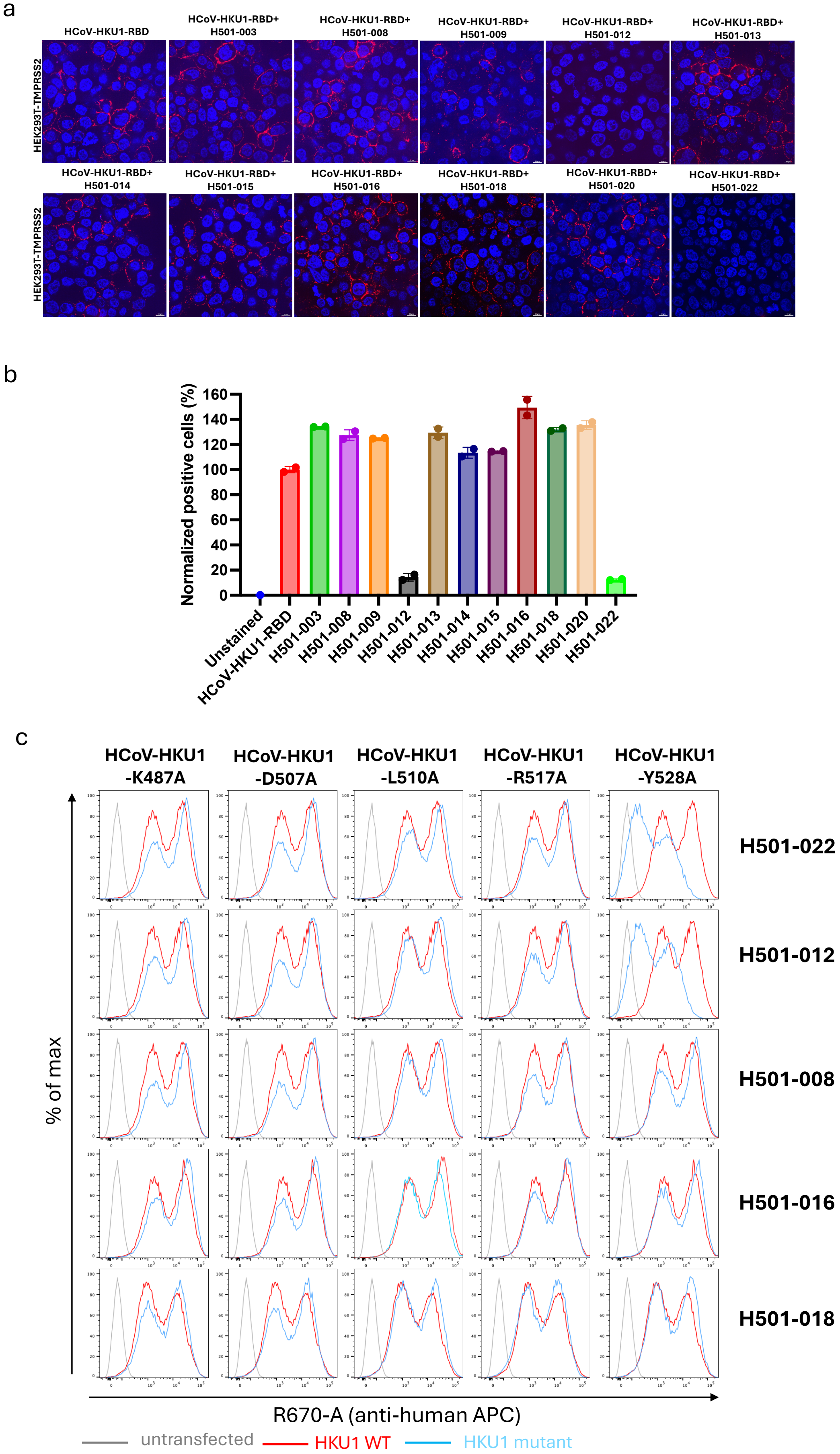
a
HCoV-HKU1-RBD
HCoV-HKU1-RBD+ H501-003
HCoV-HKU1-RBD+ H501-008
HCoV-HKU1-RBD+ H501-009
HCoV-HKU1-RBD+ H501-012
HCoV-HKU1-RBD+ H501-013
HEK293T-TMPRSS2
HCoV-HKU1-RBD+ H501-014
HCoV-HKU1-RBD+ H501-015
HCoV-HKU1-RBD+ H501-016
HCoV-HKU1-RBD+ H501-018
HCoV-HKU1-RBD+ H501-020
HCoV-HKU1-RBD+ H501-022
HEK293T-TMPRSS2
b
Normalized positive cells (%)
Unstained
HCoV-HKU1-RBD
H501-003
H501-008
H501-009
H501-012
H501-013
H501-014
H501-015
H501-016
H501-018
H501-020
H501-022
c
HCoV-HKU1 -K487A
HCoV-HKU1 -D507A
HCoV-HKU1 -L510A
HCoV-HKU1 -R517A
HCoV-HKU1 -Y528A
H501-022
H501-012
H501-008
H501-016
H501-018
% of max
R670-A (anti-human APC)
untransfected
HKU1 WT
HKU1 mutant

Extended Data Fig. 9. HCoV-HKU1 RBD-specific mAbs H501-012 and -022 block S binding to TMPRSS2. (a-b) HCoV-HKU1 RBD mAbs were analyzed for inhibition of S-TMPRSS2 interaction by immunostaining of cell surface-bound HCoV-HKU1 RBD-mAb complexes using confocal microscopy (a) and flow cytometry (b). (a) HCoV-HKU1-RBD binding with TMPRSS2 was detected using anti-human Alexa Fluor 594 (red) secondary antibodies, and the cell nucleus was stained with DAPI (blue). (b) Anti-human FITC secondary antibodies were used for flow cytometric analysis of HCoV-HKU1-RBD interaction with TMPRSS2. RBD alone binding positive cells are set to 100% and the RBD-mAb complexes are normalized to RBD alone and represented as percent positive cells. (c) H501-012 and -022 were assessed for binding to cell surface-expressed HCoV HKU1 S with indicated TMPRSS2 binding site mutations, as revealed by crystal structure in **Fig. 5**. Percentage of maximum binding to untransfected cells (gray), wild-type S-transfected cells (red), mutant S-transfected cells (light blue) are shown.

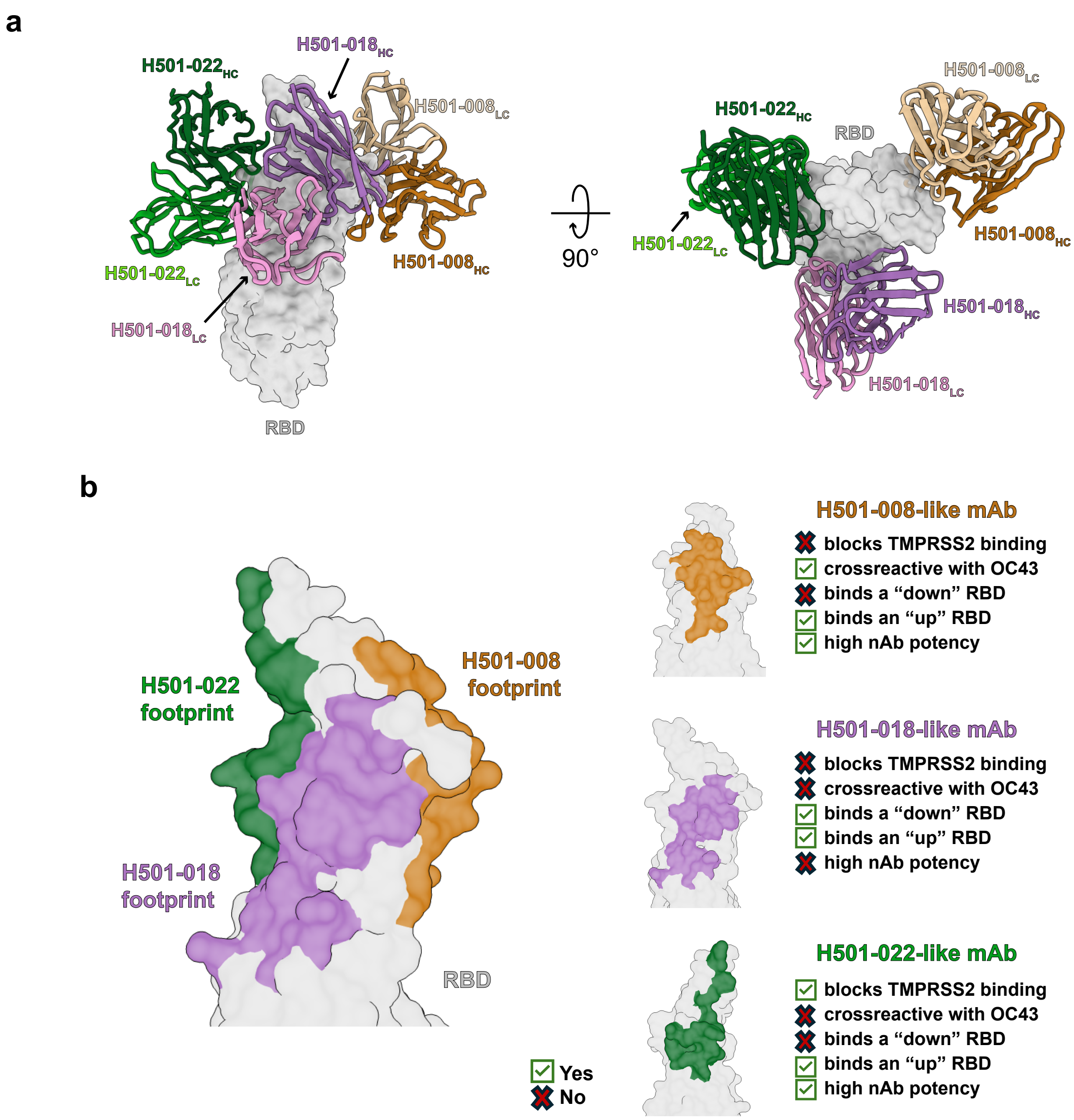

Extended Data Fig. 10. Comparisons of mAbs H501-008, -018, and -022 bound to HCoV-HKU1 RBD. (a) Heavy chains and light chains from H501-008, -018, and -022 are aligned based on the HCoV-HKU1 RBD. RBD is shown as a surface representation, and Fabs are shown as ribbon representations. (b) Summarized characteristics of H501-008, H501-018, and H501-022. RBD epitopes are color-coded based on their respective antibodies. Colors are as follows: RBD (light gray), H501-008 VH (orange), H501-008 VL (tan), H501-008 epitope (orange), H501-018

VH (purple), H501-018 VL (pink), H501-018 epitope (purple), H501-022 VH (green), H501-022 VL (light green), H501-022 epitope (green).
