# Supplementary material for: Human Coronavirus HKU1 Neutralizing Monoclonal Antibodies Target Diverse Epitopes Within and Around the TMPRSS2 Receptor Binding Site
Source: bioRxiv. 2025 Oct 30:2025.10.29.685445. Preprint. [Version 1] doi: 10.1101/2025.10.29.685445 (PMC12636560; doi:10.1101/2025.10.29.685445)
Supplement: Supplement 2 [file media-2.pdf]

Human Coronavirus HKU1 Neutralizing Monoclonal Antibodies Target Diverse Epitopes Within and Around the TMPRSS2 Receptor Binding Site

Lingshu Wang[1,16], Jeswin Joseph[2,16], Sheena Vasquez[3,16], Daniel Wrapp[4,11], Timothy P. Sheahan[5], Christian K.O. Dzuvor[2], Osnat Rosen[1,12], Robert N. Kirchdoerfer[6,13], Olubukola M. Abiona[1], Catherine Hammond[7], Wei Shi[1], Sydney P. Moak[7], Wing-Pui Kong[1], Yi Zhang[1], Michael R. Eso[3], Ariane J. Brown[5], Andrew B. Ward[6], Ralph Baric[5], Jason S. McLellan[4], Theodore C. Pierson[1], John Mascola[1,14], Barney S. Graham[1,15], Hadi M. Yassine[8], Christopher O. Barnes[3,9,10,17], Kizzmekia S. Corbett-Helaire[1,2,7,17,18]

[1]Vaccine Research Center; National Institutes of Allergy and Infectious Diseases; National Institutes of Health; Bethesda, Maryland, 20892; United States of America

[2]Department of Immunology and Infectious Diseases; Harvard T.H. Chan School of Public Health; Boston, Massachusetts, 02115; United States of America

[3]Department of Biology; Stanford University; Stanford, CA, 94305; United States of America

[4]Department of Molecular Biosciences; University of Texas at Austin; Austin, Texas, 78712; United States of America

[5]Department of Epidemiology; University of North Carolina at Chapel Hill; Chapel Hill, North Carolina, 27599; United States of America

[6]Department of Integrative Structural and Computational Biology, The Scripps Research Institute, La Jolla, CA,92037; United States of America

[7]Howard Hughes Medical Institute; Chevy Chase, Maryland, 20815; United States of America

[8]Biomedical Research Center, Member of QU Health, Qatar University; Doha, Qatar

[9]ChEM-H Institute, Stanford University; Stanford, 94305, United States of America

[10]Chan Zuckerberg Biohub; San Francisco, 94158, United States of America

[11]Current affiliation: Duke Human Vaccine Institute, Duke University School of Medicine, Durham, NC 27710; United States of America

[12]Current affiliation: Department of Biotechnology, Israel Institute for Biological Research, Ness-Ziona, Israel

[13]Current affiliation: Department of Biochemistry, Institute for Molecular Virology, Center for Quantitative Cell Imaging, University of Wisconsin-Madison, Madison, Wisconsin, 53706; United States of America

[14]Current affiliation: Modex Therapeutics, Weston, MA, 02493

[15]Current affiliation: Medicine and Microbiology, Biochemistry, & Immunology, Morehouse School of Medicine, Atlanta, GA, 30310

[16]Authors contributed equally to this study.

[17]Authors contributed equally to this study.

[18]Correspondance: kizzmekia_corbett@hsph.harvard.edu

Supplementary Figures

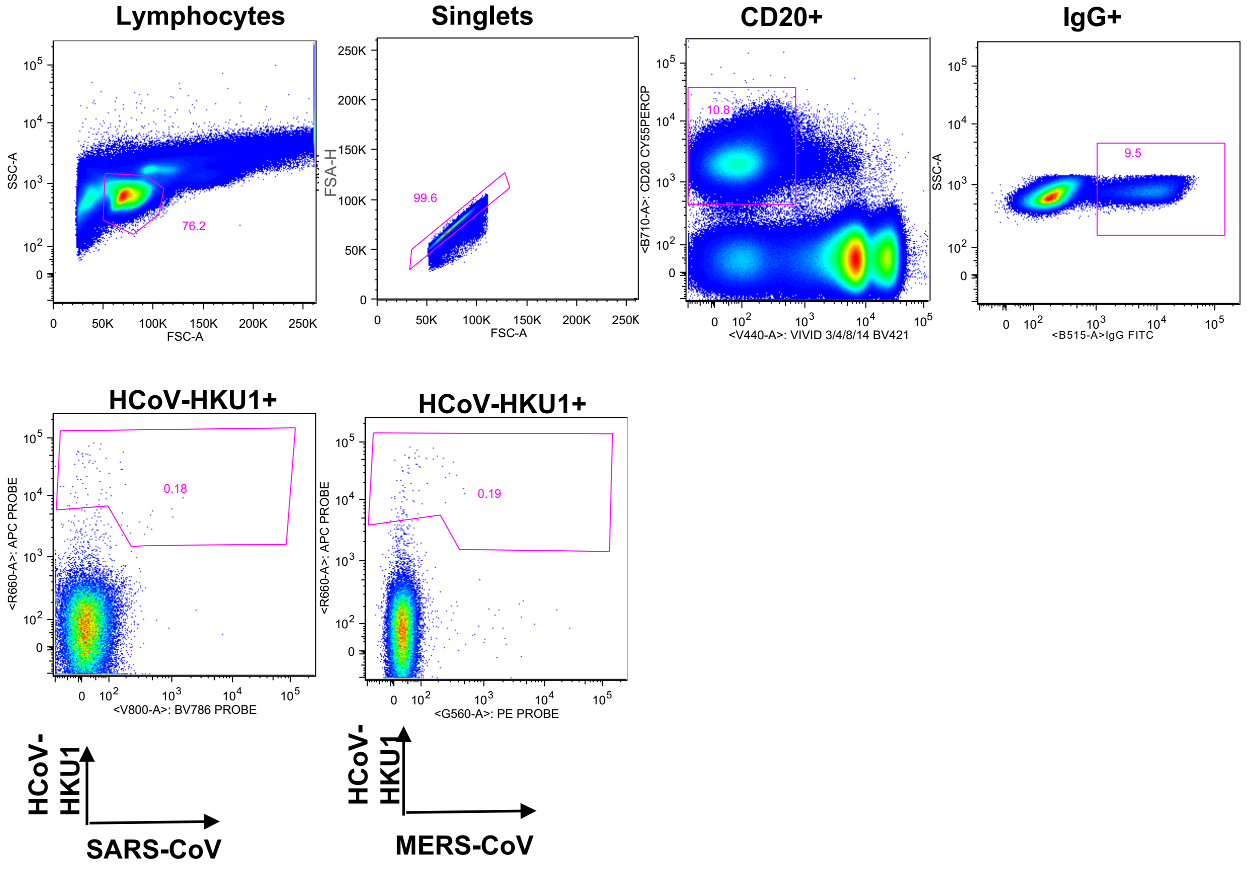

Supplementary Fig. 1. B cell sorting strategy for isolation of HCoV-HKU1 S-specific mAbs. PBMCs collected 41 days after a confirmed HCoV-HKU1 infection were stained with a viability marker (VIVID), Abs to exclude T cells and monocytes (CD3/CD4/CD8/CD14), Abs to label B cells (CD20/IgG), and S-2P probes for HCoV-HKU1, SARS-CoV, and MERS-CoV. IgG^+ HCoV-HKU1 S-$2P^+$ single B cells were sorted and subjected to PCR amplification and cloning of V_H/V_L genes. A panel of mAbs was generated and selected for further characterization based on binding specificity and neutralization potency **(Table 1)**.

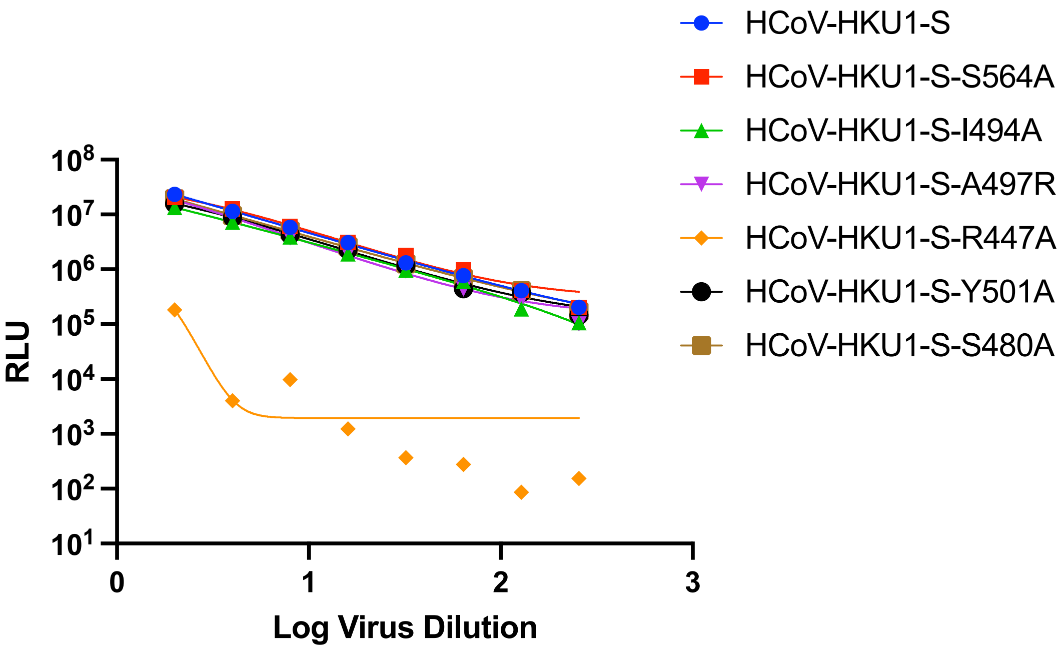

Supplementary Fig. 2. Titration of HCoV-HKU1 pseudoviruses with spike mutations. HCoV-HKU1 pseudoviruses containing indicated S RBD mutations were assessed for viral entry in HEK293T-TMPRSS2 cells. Pseudovirus entry was quantified using RLU and plotted against log virus dilutions. All mutant pseudoviruses, except HCoV-HKU1-S-R447A (orange), were infectious and their thus infectivity curves (multiple colors) overlap.

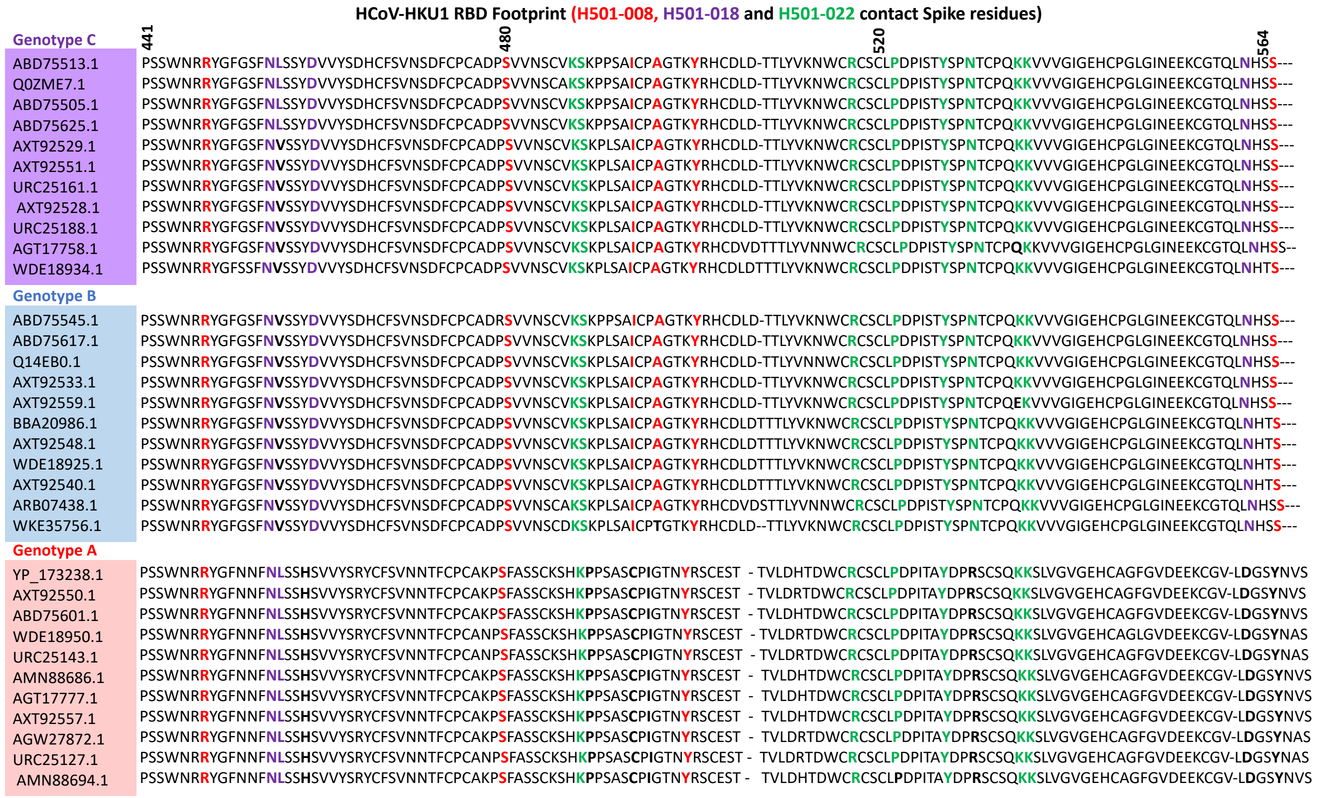

Supplementary Fig. 3. H501-008, -018, and -022 contact residues across HCoV-HKU1 strains. HCoV-HKU1 genotypes A, B, and C RBD alignment highlighting H501-008, -018, and -022 contact residues in red, purple, and green, respectively. HCoV-HKU1 S sequences were retrieved from NCBI, GenBank. In MEGA11, these sequences were aligned using the MUSCLE algorithm.

Supplementary Tables

mAbs	VH	% identity	CDRH3 length	CDRH3	VL	% identity	CDRL3 length	CDRL3
**H501-003**	IGHV4-59	95.09	19	CASAPVLGSSGPKGYYGLEVW	IGLV1-47	98.25	11	CAAWDDSLSGYVF
**H501-008**	IGHV3-30	88.89	24	CAREGPMYYDAWSGRSYYNNYALDVW	IGLV1-51	93.33	11	CGTWDSSLSAGVF
**H501-009**	IGHV4-61	92.98	15	CARSSGQIPKTEAFQHW	IGLV1-47	97.54	11	CATWDDSLRTPVF
**H501-012**	IGHV2-5	97.59	15	CVYSMSRPVAGTVIDHW	IGKV1-39	94.62	9	CQQSYSTPPTF
**H501-013**	IGHV4-61	94.74	14	CARFPGLPKTNAFDIW	IGLV1-47	95.09	11	CATWDDSLSCPVF
**H501-014**	IGHV4-59	93.33	14	CARGGFLKNSDAFDIW	IGLV1-47	95.79	11	CAAWDDSLTGPVF
**H501-015**	IGHV4-59	95.09	13	CARGSWARRLEVDNW	IGLV1-47	97.19	11	CATWDDNLGAWVF
**H501-016**	IGHV3-48	93.06	14	CATHSSNWVYDAFDIW	IGKV1-15	93.91	12	CQQYDIWPRLTF
**H501-018**	IGHV4-59	94.97	13	CARGTWSRRLEVDLW	IGLV1-47	98.60	11	CATWDDSLNSWVF
**H501-020**	IGHV4-59	93.33	14	CARGEWERRLGAFDIW	IGLV1-47	94.39	11	CSVWDDSLSAVAF
**H501-022**	IGHV2-5	96.56	15	CVYSMSRPVAGGVIDHW	IGKV1-39	93.19	9	CQQSYSSPPTF
**H501-007**	IGHV6-10	90.57	16	CARVVVRYGWNDNWFDPW	IGKV1-39	91.40	9	CQQSTGTPTF
**H501-010**	IGHV5-51	96.88	17	CARLRDEGADYYYYGMDVW	IGKV1-9	98.92	10	CQQLNSYSLGF
**H501-101**	IGHV1-46	90.62	16	CARGVDLWGNFYTYFDYW	IGKV3-20	90.07	9	CQQHDASPTF

Supplementary Table 1. mAb Heavy and light gene sequences

	HCoV-HKU1 RBD + H501-008
PDB ID	9YXW
Data collection	
Space group	$C2_1$
Cell dimensions	
a, b, c (Å)	104.5, 71.8, 174.5
α, β, γ (°)	90.0, 94.6, 90.0
Resolution (Å)	59.09-2.60 (2.71-2.60)
$I/\sigma I$	4.8 (2.2)
CC1/2	0.919 (0.703)
Completeness (%)	99.8 (99.6)
Redundancy	3.1 (3.3)
Refinement	
R_{work}/R_{free} (%)	25.2/26.7
No. atoms	
Protein	5480
Ligand	0
Water	179
Average B-factors	
Protein	116.32
Solvent	55.24
R.m.s deviations	
Bond lengths (Å)	0.009
Bond angles (Å)	1.44
Clashscore	13.54
Ramachandran	
Favored (%)	96.5
Allowed (%)	3.4
Outliers (%)	0.1

Supplementary Table 2. X-ray Crystallography data collection, refinement, and validation statistics

	HCoV-HKU1 S-2P H501-008 (EMDB-72934)	HCoV-HKU1 S-2P H501-018 (State 1) (EMDB-72935) (PDB 9YGN)	HCoV-HKU1 S-2P H501-018 (State 2) (EMDB-72936) (PDB 9YGO)	HCoV-HKU1 S-RBD H501-018 (local refine) (EMDB-72937) (PDB 9YGP)	HCoV-HKU1 S-2P H501-022 (EMDB-72938) (PDB 9YGQ)	HCoV-HKU1 S-RBD H501-022 (local refine) (EMDB-72939) (PDB 9YGR)
**Data collection and processing**						
Magnification	150000x	130000x	130000x	130000x	130000x	130000x
Voltage (kV)	200	300	300	300	300	300
Electron exposure (e–/Å^2)	40	30	30	30	30	30
Defocus range (μm)	0.7 – 2.5	0.4 – 2.5	0.4 – 2.5	0.4 – 2.5	0.4 – 2.5	0.4 – 2.5
Pixel size (Å)	0.92	0.92	0.92	0.92	0.92	0.92
Symmetry imposed	C1	C1	C1	C1	C1	C1
Initial particle images (no.)	860660	2019273	2019273	2019273	3417316	3417316
Final particle images (no.)	54647	54081	54468	141143	44565	139880
Map resolution (Å) FSC threshold 0.143 (unmasked/masked)	6.5/4.4	4.2/3.4	4.2/3.4	4.3/3.1	4.5/3.6	6.3/3.9
Map resolution range (Å)	4.1 – 12.0	3.3 – 8.3	3.2 – 4.9	3.0 – 3.6	3.4 – 8.3	3.4 – 4.5
**Refinement**						
Initial model used (PDB code)		8Y8H, 4FQK, 4QHK	8Y8G, 4FQK, 4QHK	8Y8G, 4FQK, 4QHK	8Y8I, 7T3M, 5O7P	8Y8A, 73TM, 5O7P
Model resolution (Å)						
FSC threshold 0.143		3.4	3.4	3.2	3.6	3.8
Model resolution range (Å)		3.3 - 8.3	3.2 – 4.9	3.0 – 3.6	3.4 – 8.3	3.4 – 4.5
Map sharpening *B* factor (Å^2)		51.2	55.0	61	50.6	74.9
Model composition						
Non-hydrogen atoms		32850	33115	7414	31421	3312
Protein residues		4232	4225	922	4023	432
Ligands		21	24	14	22	0
B factors (Å^2)						
Protein		101.82	75.32	79.94	112.78	67.43
Ligand		30.0	105.20	30.0	30.0	0
R.m.s. deviations						
Bond lengths (Å)		0.004	0.005	0.004	0.006	0.008
Bond angles (°)		0.932	1.076	0.925	0.977	1.093
Validation						
MolProbity score		1.86	1.89	1.70	2.10	1.85
Clashscore		7.08	8.65	4.69	8.07	7.57
Poor rotamers (%)		1.56	1.26	1.82	2.69	1.05
Ramachandran plot						
Favored (%)		95.40	94.98	96.14	95.17	93.75
Allowed (%)		4.60	5.02	3.86	4.83	6.25
Disallowed (%)		0.00	0.00	0.00	0.00	0

Supplementary Table 3. Cryo-EM data collection, refinement, and validation statistics
